# The scaling of crime concentration in cities

**DOI:** 10.1371/journal.pone.0183110

**Published:** 2017-08-11

**Authors:** Marcos Oliveira, Carmelo Bastos-Filho, Ronaldo Menezes

**Affiliations:** 1 BioComplex Laboratory, Florida Institute of Technology, Melbourne, Florida, United States of America; 2 Escola Politécnica de Pernambuco, Universidade de Pernambuco, Recife, Pernambuco, Brazil; University of Sydney, AUSTRALIA

## Abstract

Crime is a major threat to society’s well-being but lacks a statistical characterization that could lead to uncovering some of its underlying mechanisms. Evidence of nonlinear scaling of urban indicators in cities, such as wages and serious crime, has motivated the understanding of cities as complex systems—a perspective that offers insights into resources limits and sustainability, but that usually neglects details of the indicators themselves. Notably, since the nineteenth century, criminal activities have been known to occur unevenly within a city; crime concentrates in such way that most of the offenses take place in few regions of the city. Though confirmed by different studies, this concentration lacks broad analyses on its characteristics, which hinders not only the comprehension of crime dynamics but also the proposal of sounding counter-measures. Here, we developed a framework to characterize crime concentration which divides cities into regions with the same population size. We used disaggregated criminal data from 25 locations in the U.S. and the U.K., spanning from 2 to 15 years of longitudinal data. Our results confirmed that crime concentrates regardless of city and revealed that the level of concentration does not scale with city size. We found that the distribution of crime in a city can be approximated by a power-law distribution with exponent *α* that depends on the type of crime. In particular, our results showed that thefts tend to concentrate more than robberies, and robberies more than burglaries. Though criminal activities present regularities of concentration, we found that criminal ranks have the tendency to change continuously over time—features that support the perspective of *crime as a complex system* and demand analyses and evolving urban policies covering the city as a whole.

## Introduction

Cities are the fundamental drivers of human societies; their capability to bring individuals together fosters innovation, wealth creation, and economic growth, but unfortunately they suffer from problems such as pollution, disease spread, and more pervasively, crime. Yet, even though crime is a danger to the development of cities, and counter-measures are greatly desired, we still fail to understand its structure and dynamics [1, 2]. Notably, the interconnected dimensions in cities, such as social and infrastructural, coupled with their natural dynamics, requires an understanding of cities not as static objects or locations but as complex systems [3, 4]. This point of view has provided the means to comprehend the growth of cities and its impact on urban indicators, such as employment, patent, wage, and crime [5–15]. Still, only a few studies have taken into account the intricacies of these indicators when analyzing allometric relationships of cities [13–16]. For almost two centuries, however, crime in cities has been known to be unevenly distributed [17, 18]. Criminal events concentrate in such way that most of the offenses happen in very few regions [19]. Still, this aspect of crime has never been objectively characterized, albeit confirmed in different locations. Such characterization has the potential to help researchers to create realistic models of crime and to present the grounds to understand the impact of local activities on global patterns of cities.

The very notion of a city bringing people together to interact comprises the idea of emergence of self-organized coordination derived from local activities [20–23]. Despite the apparent individual disorder in the decisions and processes at local levels, cities exhibit several regularities that are argued to be a result of the need to expand and to develop [4, 23–32]. These findings support the perspective of cities as complex systems and have helped to understand various aspects of cities [6–11]. Several urban indicators have been found to scale with the population size *N* of the city according to a law of the form:

$$Y\propto N^{\beta },$$ (1)

where the exponent *β* relates to the class of the indicator [6]. For aspects associated with infrastructure (e.g., roads, gasoline stations), the quantities scale sub-linearly, while sociological dimensions, such as innovation, wealth, or crime, present superlinear scalings—though the scaling depends on the city definition and the model for Pr(*Y*|*N*) (see S1 Text) [33–35]. In the case of sublinear scalings, cities utilize resources more efficiently as they grow, while superlinear relationships imply more accumulation in larger cities. The superlinear scaling is claimed to be associated with population density and human interactions in cities [8–11]. As individuals meet in space and time, simple principles on the formation of ties can explain the existence of regularities in urban indicators, despite idiosyncrasies of each city [8]. Such models and analyses disregard, however, details of urban indicators such as variations across the city, likely due to the lack of high-granularity data. Still, social media and mobile phone data have been used to demonstrate that human interactions scale super-linearly with city size while the probability 〈*p*_*c*_〉 that two peers of an individual interact presents scale-invariance with 〈*p*_*c*_〉 ≈ 0.25 [14, 16]. Such features imply efficient spreading processes in the social network when cities grow and suggest the emergence of regularities in urban indicators as an outcome of patterns in human interactions [14].

Accordingly, human dynamics also play a major role in criminal activities, which are likely to drive patterns in crime activity [2, 36–38]. In fact, empirical evidence has shown that crime presents a remarkable regularity of concentration in several dimensions that relate to context (e.g., target, location, offender) and to features (e.g., spatial, temporal, type of crime) [39]. In particular, the spatial concentration of crime exists in such way that, regardless of granularity level, some areas have disproportionately more crime than others—popularly called hotspots [19]. The phenomenon has been confirmed in different cities using various spatial aggregation units including street and area level (e.g., street segments, census tracts, blocks) [40–42]. Such ubiquity motivated the proposition of the *law of crime concentration* which states that a small number of micro-geographic units account for most of the offenses in a neighborhood or city [19]. Yet, the use of distinct approaches to aggregate criminal events hinders an objective definition of crime concentration—though necessary to confirm the existence of the phenomenon. Even when the same type of aggregation unit is used, analyses might be biased due to particularities in the units of the cities (e.g., street segments). The lack of a more general framework for analyzing the spatial distribution of offenses prevents the general characterization of crime concentration. Such framework enables the examination of allometric scaling in cities regarding the clustering of crime and its dynamics as well as to assess signatures in different types of crime. The characterization of crime concentration paves the way for unveiling the very mechanisms that underlie the phenomenon in cities. Yet, an unbiased assessment of any regularity in crime needs to consider the relationship between population and crime, and thus an ideal framework must employ aggregation units that take into account the population in each unit [6, 12, 43–45].

Here we develop a framework to assess the distribution of criminal activities in cities by dividing the area of a city into regions with equal population size and aggregating offenses that happened within the same regions. This general framework allows us to perform a comprehensive analysis of the allometric relationship between crime distribution and city population. We examined criminal data from locations in the United States and the United Kingdom, and found that not only crime concentrates regardless of city, but also population size does not have an influence on the levels of concentration—despite the relationship between crime and total population. Crime concentration manifests in the probability distribution of crime across a city which can be described by a power law

$$p\left(x\right)\propto x^{-\alpha }$$ (2)

where the exponent *α* relates to the type of crime. From the perspective of cities as complex systems, our results indicate cities, and thus crime, growing in such a way to maintain the concentration of crime. To evaluate the dynamics of crime we measured the entropies of the ranks of criminal regions in the cities. We found that the certainty about the region in a position of the rank decreases exponentially with the position rank, which implies that we have only confidence about few of the most criminal regions of a city. The high fluctuation of crime across the city suggests that crime in cities is not in a state of equilibrium, despite the regularity in the concentration of offenses; such features support the viewpoint of *crime as a complex system*. This perspective encourages crime analyses that cover the whole city, instead of the focus on criminal hot spots. Our work sheds light on the challenges posed by the increasing number of people in cities which demands strategies towards sustainable development.

## Results

Our analysis of crime concentration is based on official disaggregated data sets of criminal occurrences from locations of different population size from the United States and the United Kingdom, summarized in Table 1. The basic information in these data sets includes the place where the offense occurred, the date when it happened, and the type of crime (e.g., burglary, theft, robbery). Here we assess the spatial concentration of crime across cities considering the regularities with respect to the concentration itself and its dynamics.

**Table 1:** Official disaggregated data sets of offenses in different locations in the U.S. and the U.K.

United States (cities)							
	Population	Period	#Records		Population	Period	#Records
Atlanta/GA	447,841	2009–2015	241,070	Los Angeles/CA	3,928,864	2012–2015	944,039
Baltimore/MD	622,104	2011–2015	261,446	New York/NY	8,550,405	2006–2015	1,123,466
Baton Rouge/LA	229,426	2011–2015	803,934	Philadelphia/PA	1,567,442	2006–2015	747,743
Boston/MA	645,966	2012–2015	268,057	Portland/OR	609,456	2004–2014	649,349
Chattanooga/TN	173,366	2011–2012	155,241	Raleigh/NC	431,746	2005–2015	492,899
Chicago/IL	2,695,598	2001–2015	6,000,707	San Francisco/CA	837,442	2003–2015	1,856,293
Dallas/TX	1,258,000	2014–2015	161,998	Santa Monica/CA	92,472	2006–2015	92,456
Denver/CO	649,495	2011–2015	366,352	Seattle/WA	652,405	2008–2015	610,079
Hartford/CT	125,017	2005–2015	516,043	St. Louis/MO	318,416	2008–2015	301,713
Kansas City/MO	467,007	2009–2015	2,679,336				
United Kingdom (police forces)							
	Population	Period	#Records		Population	Period	#Records
Cleveland	566,740	2011–2015	446,625	Leicestershire	1,005,558	2011–2015	439,950
Metropolitan	8,538,689	2011–2015	5,377,392	North Wales	687,937	2011–2015	330,527
Greater Manchester	2,732,854	2011–2015	1,701,428	West Yorkshire	2,264,329	2011–2015	1,337,565

See S1 Text for preprocessing and sources.

### Characterizing crime concentration in cities

**Fig 1:**
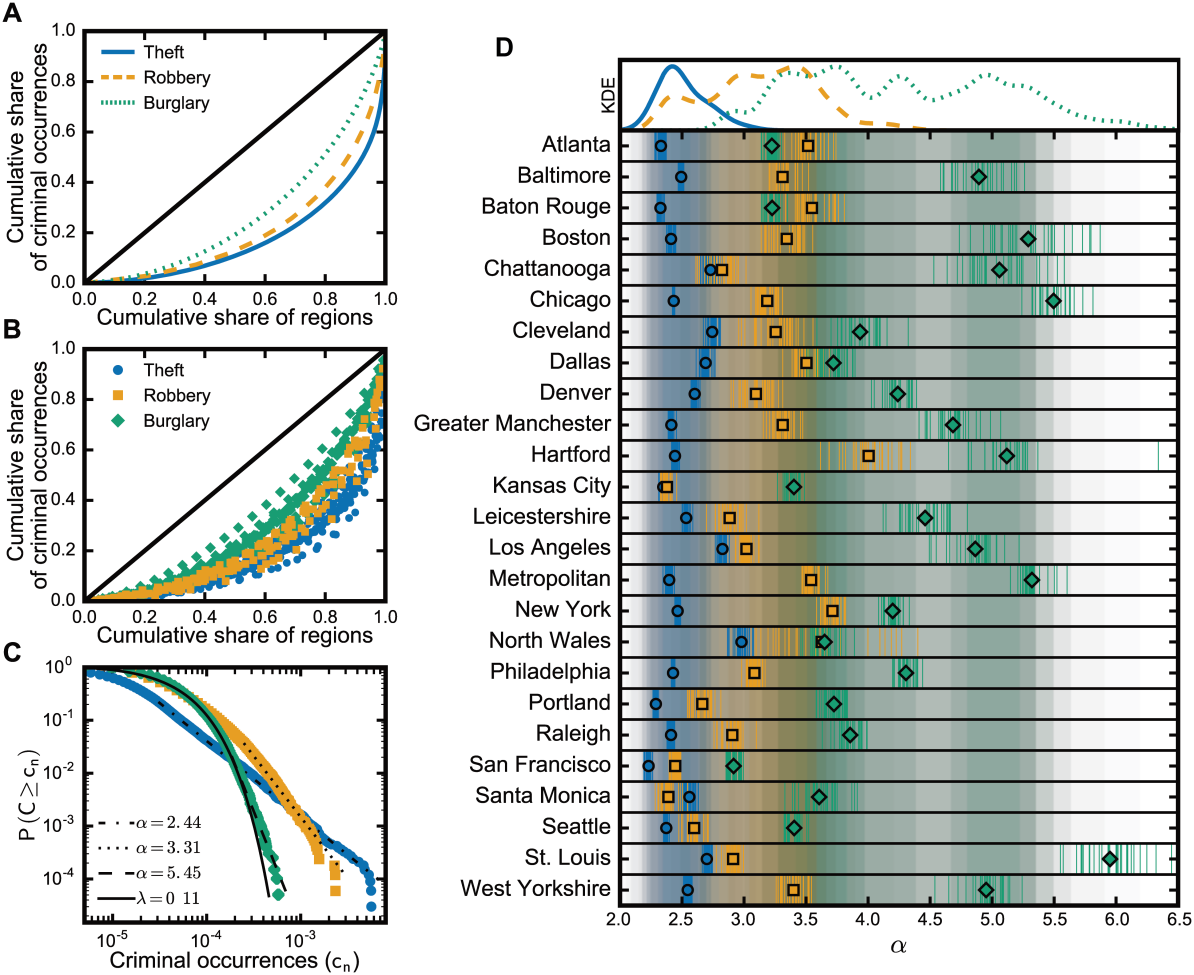
Different types of criminal activities present distinct levels of concentration in cities. (A) The Lorenz curves of the distributions of crime in the regions of Chicago reveal higher tendency of concentration in the case of thefts than in robberies and burglaries, a tendency that (B) seems to occur systematically in all considered cities: theft concentrates more than robbery, and robbery more than burglary. The difference between these types of crime manifests itself in their respective estimated complementary cumulative distribution. For instance, (C) the probability of a place with a high rate of burglaries in Chicago decays almost as fast as an exponential with λ = 0.11, while the curve for thefts follows approximately a power-law with *α* = 2.44 which decays slowly and allows the existence of places with a high number of thefts. Such pattern of concentration occurs similarly (D) in the other cities (circles, squares, and diamonds, are the means from each set of arrangements) in which the exponents for theft seem to be well-behaved in the interval [2, 3], whereas robbery and burglary have wider ranges (actual values are found in S1 Text), as depicted by the density estimation (KDE using Gaussian basis with *h* = 0.2).

We divided each city into regions with the same population size and analyzed the distribution of the number offenses that occurred within each region. When dividing a region into areas with the same population size, it is important to understand that there are a very large possible number of divisions. Hence, for each city *c* we first generated 30 arrangements in which each comprises of *R*_*c*_ same-population divisions of the city (see Methods), then aggregated the occurrences of crime by type of crime such as theft, burglary, and robbery; the aggregation was done for each arrangement. Such procedure provides us a generalized approach to assess the distribution of crime in a city by examining the number of occurrences across regions. For instance, the Lorenz curves of crimes in Chicago, depicted in Fig 1A, show that the distribution of crime in the city seems not only to concentrate but also to present different levels of concentration that depend on the type of crime. In fact, as shown in Fig 1B, all considered cities appear to exhibit similar patterns: thefts concentrate more than robberies, and robberies concentrate more than burglaries.

To assess the regularities in the concentration of crime, we fit the distribution of crime in each arrangement with the following distributions: power law, truncated power law, lognormal, exponential, and stretched exponential; and then compare them using the likelihood ratio test [46]. For each arrangement, we tested the plausibility of the power law to describe the crime distribution and compare the fits against the alternatives. We performed this procedure on all arrangement for all types of crime in all considered cities in order to give a score to each model for each city–crime pair. In most of the data sets, we found moderate support to the power-law distribution; from the 75 city–crime pairs, the truncated power law was favored only in 4 cities when taking into account thefts, 5 in the case of robberies, and 2 cities for burglary data (details in Splitting cities). By analyzing the estimated *α* of the pure power-law fits, we found that the exponent value relates to the type of crime. For instance, the estimated exponents of the power law for the distribution of crime in Chicago (Fig 1C) yield *α*_*t*_ ≈ 2.44 for theft, *α*_*r*_ ≈ 3.31 for robbery, and *α*_*b*_ ≈ 5.45 for burglary—in agreement with the Lorenz curves given that higher values for *α* imply lower likelihood of concentrated criminal spots. Our results revealed that different types of crime present distinct levels of concentration which manifests on the range of the power-law exponent: *α*_*t*_ is between 2.1 and 3.0; whereas the exponents for burglaries *α*_*b*_ and robberies *α*_*r*_ vary in wider ranges with *α*_*r*_ within 2.4 and 4.1, and *α*_*b*_ between 2.9 and 6.0 (see Fig 1D). Note that, in some data sets from the ones that exhibit large *α* values, we found that the exponential and power-law distributions are both good descriptions of the data, albeit the power law describing better crime distribution when small *α* values (see S1 Text). The distinct exponent intervals are plausibly due to particularities in the dynamics of each type of crime. The well-behaved interval of *α*_*t*_ suggests independence of the dynamics of theft from the idiosyncrasies of the cities; whereas the high variance of *α*_*r*_ and *α*_*b*_ suggests a dependency on the characteristics of the city, such as city layout, demographics. Despite the differences between exponents, our results showed that *α*_*t*_ ≤ *α*_*r*_ ≤ *α*_*b*_ in all the cities with the exceptions of Santa Monica (*α*_*r*_ < *α*_*t*_), Baton Rouge and Atlanta (*α*_*b*_ < *α*_*r*_). Though the regions in the cities have the same population size, the distributions of crime in the regions are highly skewed and depend on crime type.

**Fig 2:**
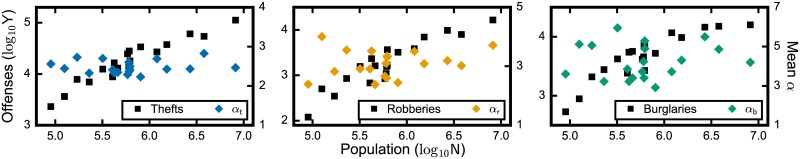
The size of the city lacks influence on the level of crime concentration. Though the growth of a city implies an increase in crime rates, the spatial concentration of offenses seems to be independent of the population size of the city. To test this, we employed Hoeffding’s independence test from which we could not reject the hypothesis that population size and the exponent of the power-law fit of the crime distribution are independent. In the case of thefts, the well-behaved *α* exponent suggests scale invariance, while robberies and burglaries seem to be more sensible to the cities, albeit uncorrelated with city size. In the double-y-axes plots, squares indicate the total number of offenses in a city during a year; while diamonds represent the average power-law exponent for a city.

The allometric scaling of crime in cities suggests, however, a similar relationship between the concentration of crime and population size. To examine the relationship we evaluate the statistical dependence between city size and the distribution of crime across the city. We employ the Hoeffding’s test of independence H between the population size of the cities and the average power-law exponent *α*. We here analyze the U.S. urban system and thus use the census data from the considered U.S. cities and the estimated power-law exponents found for each type of crime in the city (see Fig 2). From our experiments, we could not reject the hypothesis that the size of the city and the level of crime concentration are independent with the 95% confidence. The disassociation found between the distribution of crime and the system size indicates crime concentration as an attribute of criminal phenomena which occurs regardless of the population size of the city; that is, not only crime concentrates, but also this concentration is not related to the size of the city, despite the existence of population and crime relationship.

### The entropy of crime concentration

**Fig 3:**
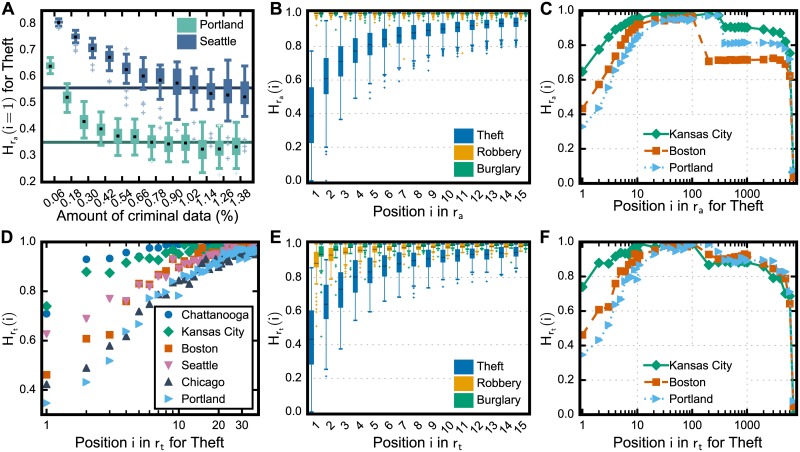
Crime moves across the regions in the cities. Though criminal activities exhibit regularities in their spatial concentration, the relative amount of crime in the regions of the city changes continuously over time. For that, we calculated the Shannon entropy of the positions in the criminal ranks of regions *r*_*t*_ and *r*_*a*_ which are created using the number of offenses aggregated by time and by the total amount of crime, respectively. In the case of *r*_*a*_, we used data slices of size that (A) minimizes the entropy of the first position of the rank in order to measure (B–C) the entropies of the positions in the rank for all considered U.S. cities. For the time-based rank, weekly data allowed us to measure (D–F) the entropies with respect to time. The overall high entropy in the positions of both ranks indicates that crime is likely to fluctuate across the city, leading to uncertainty about the regions in the rank; still, the most criminal regions have the tendency to be the same ones.

To assess the dynamics of crime, we measure the entropy of the positions in the rank of criminal spots over time. We thus divide each data set in temporal intervals using two procedures: amount-based and time-based. In the former, data is aggregated every *a*_*w*_ records; whereas the latter aggregates data every *t*_*w*_ days. The two approaches are used to take into account possible discrepancies in crime dynamics due to the existence of cities with high and low crime rates. To analyze the relative variation of crime in a given city, we first rank its regions by the amount of crime using each instance of aggregation *w* that is created by the amount-based and time-based approaches, which results in the ranks $r_{a}^{w}$ and $r_{t}^{w}$. The instances of the ranks over time allow us to measure the entropy of the position *i* in the rank as $H_{r}^{c}\left(i\right)=-\sum_{s=1}^{R_{c}}p_{i}\left(s\right)\log p_{i}\left(s\right)$ where *p*_*i*_(*s*) is the probability that the region *s* is in the *i*th position of the rank *r* of the city *c* (see S1 Text). Here we separately evaluated $H_{r_{a}}^{c}$ and $H_{r_{t}}^{c}$ for the ranks *r*_*a*_ and *r*_*t*_ of each considered city and type of crime. We used two-years data to enable us to compare the considered cities given that the smallest longest interval of longitudinal data among all cities is two years. In the case of *r*_*t*_, we aggregated data every *t*_*w*_ = 7 which allows us to capture weekly variation and guarantees enough number of instances of aggregation to calculate the probabilities. For the amount-based approach, we constructed *r*_*a*_ for each city by aggregating every $a_{w}=a_{w}^{c}$ records, where $a_{w}^{c}$ is the value at which the entropy stabilizes on a minimum value (see Fig 3A).

**Fig 4:**
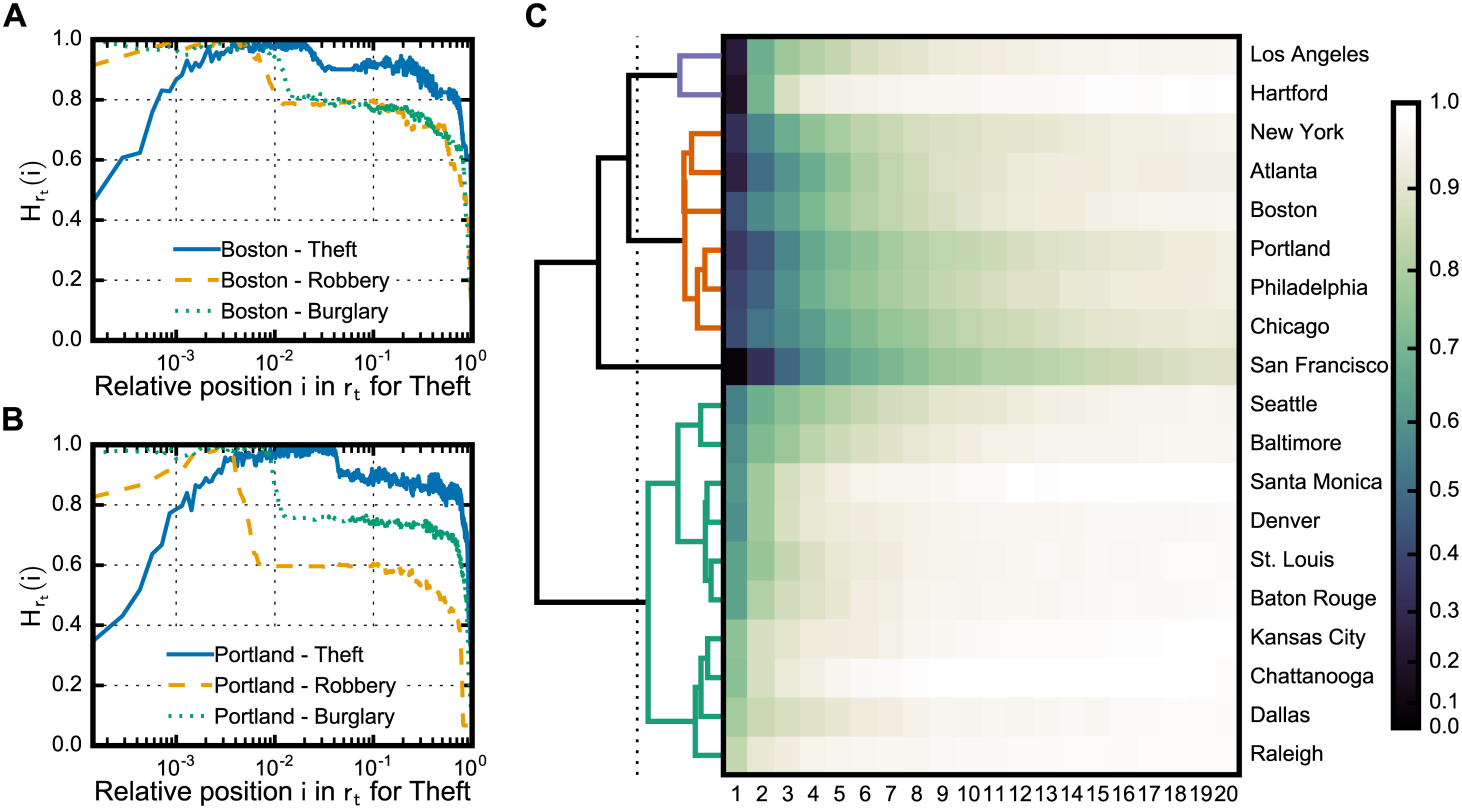
The categories of criminal regions and the categories of crime dynamics in cities. The entropy in the ranks of a given city (A–B) increases rapidly with position, reaching a peak in which the uncertainty about the regions in this interval of positions is the highest for the particular city. After this range of minimal information, the entropy drops to an interval of steady entropy, then finally decreases to zero entropy. The intervals of increasing, highest, and steady, can be seen as different categories of regions in the criminal ranks. The steady-entropy positions vanish when the ranks are created with unstable sorting algorithms, which means that these positions hold criminal regions with a similar number of offenses. Not only regions but also (C) some cities present similar dynamics of crime—as also seen in Fig 3D. Cities group together in three distinct categories with respect to dynamics of theft: stable hottest spot, stable hot spots, and less stable hot spots. Here we used hierarchical clustering with Euclidean distance and define 0.5 as the threshold to segment clusters.

We found that most positions in both ranks tend to have high entropy with sample means ${\bar{H}}_{r}=\sum_{c}\sum_{i}H_{r}^{c}\left(i\right)/R_{c}/N$ among the cities for thefts ${\bar{H}}_{r_{a}}=0.98$ and ${\bar{H}}_{r_{t}}=0.97$, ${\bar{H}}_{r_{a}}=0.99$ and ${\bar{H}}_{r_{t}}=0.95$ for burglaries, and ${\bar{H}}_{r_{a}}=0.98$ and ${\bar{H}}_{r_{t}}=0.96$ for robberies, which indicates that criminal spots are likely to vary across regions over time (see Fig 3B and 3E). Still, the first positions in the rank present distinct dynamics with the entropy *H*(*i*) of a position *i* in both ranks increasing quickly with the position *i* which means that the most criminal places have the tendency to be the same regions. In particular, our results revealed that the rank of thefts present lower entropy in the first rank positions in comparison to the other types of crime, and we found that *H*(*i*) reaches its highest value when *i* > 10 for *r*_*t*_, as seen in Fig 3D for some cities, and when *i* > 15 for *r*_*a*_. In other words, we have more certainty about the whereabouts of the hottest spots of theft than the hottest spots of robbery and burglary. Similarly, our results showed that the regions with few number of crime are usually the same ones. As depicted in Fig 3C and 3F, the entropy rapidly increases with the position of the rank, reaching the peak of uncertainty, then the values decrease to a range of positions with steady entropy. In order to examine this steady range, we analyzed ranks that are constructed with stable and unstable sorting algorithms. The rationale here is to evaluate the influence of ties in the rank on the entropy: unstable sorting gives different ranks in the case of ties and thus increasing the entropy of the positions. We found that unstable ranks result in non-decreasing entropy, which implies that the steady entropy range is due to ties in the rank. The drops in the curves are due to regions with a similar number of crime over time, a behavior also observed in the other types of crime (see Fig 4A and 4B). Still, the values of the entropies decrease to zero in the last positions of the rank, which represent regions where a crime was never recorded. Note that this procedure can help us to identify categories of regions in the city with respect to the dynamics of crime.

Not only categories of regions, we also found categories of cities. The curves of $H_{r_{t}}$ seen in Fig 3D suggest that some cities present similar dynamics in the hottest spots of theft. To examine such similarities, we employed hierarchical clustering for building clusters of cities according to their ranks; we used the entropies of the first 20 positions in their rank as the feature vector for each city (see S1 Text). Our results revealed three distinct groups in which cities have (*i*) stable hottest spot (e.g., Los Angeles, Hartford), (*ii*) stable hot spots (e.g., Chicago, Atlanta), and (*iii*) less stable hot spots (e.g., Dallas, Seattle), as illustrated in Fig 4C. Such categories arise from the signatures in the dynamics of criminal regions in cities and describe the relative crime mobility in a city (i.e., changes in the ranks). Though criminal activities concentrate regardless of city, crime continuously flows across the city, and some cities present similar dynamics in the most criminal regions.

## Discussion

Crime is ubiquitous in cities but needs still quantitative understanding. To characterize crime in cities, we examined criminal activities in 25 locations from two different countries using longitudinal data sets spanning 2 to 15 years. We developed a method to assess the spatial concentration of crime which divides a city into regions based on the resident population; then analyzed the distribution of crime in the regions. In all considered cities, we were able to confirm previous studies and identified that offenses take place in few regions of a city. Here we performed a comprehensive statistical characterization of the phenomenon in cities and showed that not only crime concentrates but also presents concentration level that depends on the type of crime and exhibits independence of the size of the city—despite the relationship between population and number of crimes. Yet, though cities have such regularity in the concentration of crime, our results revealed that criminal ranks in the cities have the tendency to change over time.

The regularities in the concentration of crime coupled with the constant displacement of crime suggest an understanding of crime as a complex system. Criminal activities flow continuously across the city while maintaining the organization of the system in such way that its dynamics and regularities appear to be scale-invariant. Different types of crime exhibit particular dynamics that lead to distinct levels of concentration and allometric scaling laws. Our results revealed thefts presenting a well-behaved concentration over cities which indicates invariance with city size and with idiosyncrasies of cities; while burglaries and robberies are more dependent on the city. These findings are particularly intriguing in light of the superlinear scaling found in thefts in contrast to the linearity in burglaries—though we are still in need of more conclusive analyses on the scaling laws of robberies (see S1 Text). Such regularities in crime concentration might be linked to the way crime scales in cities.

The characterization of crime paves the way for a better understanding of crime dynamics and provides the means to create and validate models. Though the proposal of a generative mechanism is beyond the scope of the present study, our framework can be employed for modeling given its implicit network of regions which can be used to represent a city. A theory or model attempting to explain this complex phenomenon have to conform to the skewed distribution of crime and the existence of distinct concentrations of offenses for different types of crime. For instance, models for burglary are expected to be more dependent on features of the city such as the layout of the streets or demographics. One should not conclude that we argue for any universality of power laws here, but instead we present statistical characteristics in criminal activities which we systematically found in different locations [47].

The perspective of crime as a complex system demands analyses that need to cover the system as a whole in order to assess crime. The connectedness of the city suggests that one should resist to neglect the “cold” areas by studying solely the hotspots of crime. Moreover, our results suggest that areas of high concentration of crime are expected to exist as the city grows—finding that urges for proper government policies. Still, the notion of the city as a process implies that developing static policies is likely to fail and, as such, policy-makers should pursue evolving strategies based on real-time data [48]. Urban planners may take advantage of our framework to analyze different types of criminal regions and categories of crime dynamics. Such objective analyses of the city have the potential to assist sustainable urban development, not only regarding crime but also with respect to other demographics.

## Methods

### Data sources

Since police departments employ different nomenclature for types of crime as well as different subcategory of offenses, we preprocessed the records in order to group together thefts, burglaries, and robberies (as described in S1 Text). For the spatial analysis, we considered the bounding box of the U.S. cities and the jurisdiction of the U.K. constabularies. In the case of the temporal analysis, we analyzed only the U.S. cities because the U.K. data include solely the month when offenses occurred. The sources for all the criminal data sets and census bases are further described in S1 Text.

### Splitting cities

To split a city into regions with same population size, we use census data in order to build a graph with nodes that represents roughly the same number of people and divide this graph into *R* partitions. To construct the graph, a set *s*_*i*_ of *p*_*i*_ random coordinates is created for each census block *b*_*i*_ of a place *L*, where *p*_*i*_ is the number of people in *b*_*i*_ and each x–y coordinate is uniformly generated within the geographical shape of the block. The nodes of the graph are created based on the cells of each Voronoi diagram *v*_*i*_ that is constructed from each *s*_*i*_, and the edges between nodes exist if their respective cells are neighbors of each other. Finally, this graph can be partitioned using a graph partitioning algorithm in order to generate regions (i.e., partitions) with approximately the same population size [49]. Still, to properly analyze crime in a given city *c* with this method, a value for *R*_*c*_ has to be chosen to allow us to examine crime distribution. In all data sets we analyzed, we found that the number of regions that contain at least one offense *R*^*n* ≥ 1^ increases with the total number of regions *R*, until *R*^*n* ≥ 1^ saturates at a point *R*^*n* ≥ 1^(*r*_*u*_) = *u* in which new regions do not have any crime occurring within them. A plausible reason for such behavior is the accuracy level used in police offices as offenses are registered in the criminal systems. In order not to bias our results with any particularity of such procedures, we have to set *R*_*c*_ = *ρr*_*u*_ with *ρ* lesser than the unit and sufficiently high to avoid any averaging problem [50], thus for all data sets, we define *ρ* = 0.9 (see S1 Text).

## Supporting information

S1 Text Supplementary material. (PDF)

## References

[1] Kates RW, Parris TM. Long-term trends and a sustainability transition. Proceedings of the National Academy of Sciences. 2003;100(14):8062–8067. 10.1073/pnas.1231331100 PMC166182 12829798

[2] D’Orsogna MR, Perc M. Statistical physics of crime: A review. Physics of Life Reviews. 2015;12:1–21. 10.1016/j.plrev.2014.11.001 25468514

[3] Batty M. New ways of looking at cities. Nature. 1995;377(6550):574–574. 10.1038/377574a0

[4] Batty M. The size, scale, and shape of cities. Science. 2008;319(5864):769–771. 10.1126/science.1151419 18258906

[5] Batty M. A Theory of City Size. Science. 2013;340(6139):1418–1419. 10.1126/science.1239870 23788792

[6] Bettencourt LMA, Lobo J, Helbing D, Kuhnert C, West GB. Growth, innovation, scaling, and the pace of life in cities. Proceedings of the National Academy of Sciences. 2007;104(17):7301–7306. 10.1073/pnas.0610172104 PMC1852329 17438298

[7] Bettencourt L, West G. A unified theory of urban living. Nature. 2010;467(7318):912–913. 10.1038/467912a 20962823

[8] Bettencourt LMA. The origins of scaling in cities. Science. 2013;340(6139):1438–1441. 10.1126/science.1235823 23788793

[9] Arbesman S, Kleinberg JM, Strogatz SH. Superlinear scaling for innovation in cities. Physical Review E. 2009;79(1):016115 10.1103/PhysRevE.79.016115 19257115

[10] Pan W, Ghoshal G, Krumme C, Cebrian M, Pentland A. Urban characteristics attributable to density-driven tie formation. Nature communications. 2013;4:1961 10.1038/ncomms2961 23736887

[11] Yakubo K, Saijo Y, Korošak D. Superlinear and sublinear urban scaling in geographical networks modeling cities. Physical Review E—Statistical, Nonlinear, and Soft Matter Physics. 2014;90(2):1–10. 10.1103/PhysRevE.90.022803 25215777

[12] Hanley QS, Lewis D, Ribeiro HV. Rural to Urban Population Density Scaling of Crime and Property Transactions in English and Welsh Parliamentary Constituencies. PLOS ONE. 2016;11(2):e0149546 10.1371/journal.pone.0149546 26886219 PMC4757021

[13] Bettencourt LMA, Samaniego H, Youn H. Professional diversity and the productivity of cities. Scientific Reports. 2014;4:3–8. 10.1038/srep05393 PMC4066264 24953448

[14] Schläpfer M, Bettencourt LMa, Grauwin S, Raschke M, Claxton R, Smoreda Z, et al The scaling of human interactions with city size. Journal of the Royal Society, Interface / the Royal Society. 2014;11(98):20130789–. 10.1098/rsif.2013.0789 PMC4233681 24990287

[15] Caminha C, Furtado V, Pequeno THC, Ponte C, Melo HPM, Oliveira EA, et al Human mobility in large cities as a proxy for crime. PLOS ONE. 2017;12(2):e0171609 10.1371/journal.pone.0171609 28158268 PMC5291516

[16] Tizzoni M, Sun K, Benusiglio D, Karsai M, Perra N. The Scaling of Human Contacts and Epidemic Processes in Metapopulation Networks. Scientific Reports. 2015;5:15111 10.1038/srep15111 26478209 PMC4609962

[17] Balbi A, Guerry AM. Statistique comparée de l’état de l’instruction et du nombre des crimes dans les divers arrondissements des Académies et des Cours Royales de France; 1829. Jules Renouard.

[18] Quetelet A. Recherches sur le penchant au crime aux différens âges. Brussels: M. Hayez; 1833.

[19] Weisburd D. The law of crime concentration and the criminology of place. Criminology. 2015;53(2):133–157. 10.1111/1745-9125.12070

[20] Jacobs J. The Death and Life of Great American Cities Vintage Books; 1961.

[21] Batty M, Xie Y. Self-organized criticality and urban development. Discrete Dynamics in Nature and Society. 1999;3(2-3):109–124. 10.1155/S1026022699000151

[22] Portugali J. Self-Organization and the City Springer Series in Synergetics. Berlin, Heidelberg: Springer Berlin Heidelberg; 2000.

[23] Batty M. Building a science of cities. Cities. 2012;29:S9–S16. 10.1016/j.cities.2011.11.008

[24] Batty M. The New Science of Cities. MIT Press; 2013.

[25] Bettencourt LMA, Lobo J, Strumsky D. Invention in the city: Increasing returns to patenting as a scaling function of metropolitan size. Research Policy. 2007;36(1):107–120. 10.1016/j.respol.2006.09.026

[26] Batty M, Carvalho R, Hudson-Smith A, Milton R, Smith D, Steadman P. Scaling and allometry in the building geometries of Greater London. The European Physical Journal B. 2008;63(3):303–314. 10.1140/epjb/e2008-00251-5

[27] Samaniego H, Moses ME. Cities as Organisms: Allometric Scaling of Urban Road Networks. Journal of Transport and Land Use. 2008;1(1):21–39. 10.5198/jtlu.v1i1.29

[28] De Long JP, Burger O. Socio-economic instability and the scaling of energy use with population size. PLoS ONE. 2015;10(6):1–12. 10.1371/journal.pone.0130547 PMC4474831 26091499

[29] Schiff N. Cities and product variety: evidence from restaurants. Journal of Economic Geography. 2015;15(6):1085–1123. 10.1093/jeg/lbu040

[30] Bettencourt LMA, Lobo J. Urban scaling in Europe. Journal of The Royal Society Interface. 2016;13(116):20160005 10.1098/rsif.2016.0005 PMC4843676 26984190

[31] Youn H, Bettencourt LMA, Lobo J, Strumsky D, Samaniego H, West GB. Scaling and universality in urban economic diversification. Journal of the Royal Society, Interface / the Royal Society. 2016;13(114):20150937–. 10.1098/rsif.2015.0937 PMC4759798 26790997

[32] Van Raan AFJ, Van Der Meulen G, Goedhart W. Urban scaling of cities in the Netherlands. PLoS ONE. 2016;11(1):1–16. 10.1371/journal.pone.0146775 PMC4708983 26751785

[33] Gomez-Lievano A, Youn H, Bettencourt LMA. The Statistics of Urban Scaling and Their Connection to Zipf’s Law. PLoS ONE. 2012;7(7):e40393 10.1371/journal.pone.0040393 22815745 PMC3399879

[34] Louf R, Barthelemy M. Scaling: Lost in the Smog. Environment and Planning B: Planning and Design. 2014;41(5):767–769. 10.1068/b4105c

[35] Leitão JC, Miotto JM, Gerlach M, Altmann EG. Is this scaling nonlinear? Royal Society Open Science. 2016;3(7):150649 10.1098/rsos.150649 27493764 PMC4968456

[36] White S, Yehle T, Barbosa Filho HS, Oliveira MA, Menezes R. The Spatial Structure of Crime in Urban Environments In: SocInfo Workshops. Springer; 2014 p. 102–111.

[37] Oliveira M, Barbosa-Filho H, Yehle T, White S, Menezes R. From criminal spheres of familiarity to crime networks In: Complex Networks VI. Springer; 2015 p. 219–230.

[38] Alves LGA, Lenzi EK, Mendes RS, Ribeiro HV. Spatial correlations, clustering and percolation-like transitions in homicide crimes. EPL (Europhysics Letters). 2015;111(1):18002 10.1209/0295-5075/111/18002

[39] Farrell G. Crime concentration theory. Crime Prevention & Community Safety. 2015;17(4):233–248. 10.1057/cpcs.2015.17

[40] Eck E J, Clarke RV, Guerette RT. Risky Facilities: Crime Concentration in Homogeneous Sets of Establishments and Facilities In: Farrell G, Bowers KJ, Johnson SD, Townsley M, editors. Imagination for Crime Prevention: Essays in honour of Ken Pease. vol. 21 Monsey, NY, USA: Criminal Justice Press; 2007 p. 225–264.

[41] Johnson SD. A brief history of the analysis of crime concentration. European Journal of Applied Mathematics. 2010;21(4-5):349–370. 10.1017/S0956792510000082

[42] Braga AA, Papachristos AV, Hureau DM. The Concentration and Stability of Gun Violence at Micro Places in Boston, 1980–2008. Journal of Quantitative Criminology. 2010;26(1):33–53. 10.1007/s10940-009-9082-x

[43] Alves LGA, Ribeiro HV, Mendes RS. Scaling laws in the dynamics of crime growth rate. Physica A: Statistical Mechanics and its Applications. 2013;392(11):2672–2679. 10.1016/j.physa.2013.02.002

[44] Alves LGA, Ribeiro HV, Lenzi EK, Mendes RS. Distance to the Scaling Law: A Useful Approach for Unveiling Relationships between Crime and Urban Metrics. PLoS ONE. 2013;8(8):e69580 10.1371/journal.pone.0069580 23940525 PMC3734155

[45] Hanley QS, Khatun S, Yosef A, Dyer RM. Fluctuation Scaling, Taylor’s Law, and Crime. PLoS ONE. 2014;9(10):e109004 10.1371/journal.pone.0109004 25271781 PMC4182799

[46] Clauset A, Shalizi CR, Newman MEJ. Power-Law Distributions in Empirical Data. SIAM Review. 2009;51(4):661–703. 10.1137/070710111

[47] Stumpf MPH, Porter MA. Critical Truths About Power Laws. Science. 2012;335(6069):665–666. 10.1126/science.1216142 22323807

[48] Bettencourt LMA. Cities as complex systems In: Furtado BA, Sakowski PAM, Tovolli MH, editors. Modeling Complex Systems for Public Policies. 1st ed Institute for Applied Economic Research; 2015 p. 396.

[49] Sanders P, Schulz C. Think Locally, Act Globally: Highly Balanced Graph Partitioning. In: Proceedings of the 12th International Symposium on Experimental Algorithms (SEA’13). vol. 7933 of LNCS. Springer; 2013. p. 164–175.

[50] Weisburd D, Bruinsma GJN, Bernasco W. Units of Analysis in Geographic Criminology: Historical Development, Critical Issues, and Open Questions In: Putting Crime in its Place. New York, NY: Springer New York; 2009 p. 3–31.

