# Supplementary material for: The scaling of crime concentration in cities
Source: PLoS One. 2017 Aug 11;12(8):e0183110. doi: 10.1371/journal.pone.0183110 (PMC5553724; doi:10.1371/journal.pone.0183110)
Supplement: S1 Text — (PDF) [file pone.0183110.s001.pdf]

The Scaling of Crime Concentration in Cities
Supplementary Material

Marcos Oliveira[1*], Carmelo Bastos-Filho[2], Ronaldo Menezes[1]

1 BioComplex Laboratory, Florida Institute of Technology, Melbourne, Florida, USA
2 Universidade de Pernambuco, Recife, Pernambuco, Brazil
* `moliveira@biocomplexlab.org`

Contents

1 Data **1**
1.1 Criminal events . . . 1
1.2 Geospatial information . . . 7
1.3 Population . . . 8

2 Splitting cities **8**
2.1 The number of splits and the analysis of crime . . . 8
2.2 Arrangements . . . 9

3 Model **10**

4 Independence test for alpha vs population **12**

5 Scaling laws of crime **13**

6 Entropy of rank **14**
6.1 Clustering cities . . . 14

1 Data

We carried out our analysis using official data of cities with respect to (*i*) their criminal activities, (*ii*) their geography, and (*iii*) their distribution of resident population. Here we provide the sources of the data and summarize the preprocess step performed in order to implement our experiments.

1.1 Criminal events

We used data sets of criminal occurrences in disaggregated level that contains the longitude and latitude of each offense. We obtained this data from 19 cities from United States and 6 police forces (constabularies) from United Kingdom. In the case of UK, the data was acquired from the UK Government website[1] that makes available data of crime every month since 2010 which is published by the UK Home Office. The data of crime in U.S. cities were retrieved from the respective police offices of each considered city via their websites that are described in Table 1.

Although the aforementioned data sets have their own particularities, each criminal event in any of them is characterized by the following:

- `type` - the category of the criminal event;
- `address` - the address where the crime occurred;
- `location` - the latitude and longitude where the crime occurred.

[1] `https://data.police.uk/`

Table 1: Data source for each considered U.S. city

City	Crime data source
Atlanta	http://www.atlantapd.org/crimedatadownloads.aspx
Baltimore	https://data.baltimorecity.gov/Public-Safety/BPD-Part-1-Victim-Based-Crime-Data/wsfq-mvij/data
Baton Rouge	https://data.brla.gov/Public-Safety/Baton-Rouge-Crime-Incidents/fabb-cnnu
Boston	https://data.cityofboston.gov/Public-Safety/Crime-Incident-Reports/7cdf-6fgx
Chattanooga	https://data.chattlibrary.org/Government/Crime-Data/5na4-ggsr
Chicago	https://data.cityofchicago.org/Public-Safety/Crimes-2001-to-present/ijzp-q8t2
Dallas	https://www.dallasopendata.com/browse?category=Police&limitTo=datasets
Denver	https://www.denvergov.org/opendata/dataset/city-and-county-of-denver-crime
Hartford	https://data.hartford.gov/Public-Safety/Police-Incidents-01012005-to-Current/889t-nwfu
Kansas City	https://data.kcmo.org/browse?q=crime&Type=[object%20Object]&sortBy=relevance&utf8=%E2%9C%93
Los Angeles	https://data.lacity.org/A-Safe-City/Crimes-2012-2015/s9rj-h3s6/data
New York	https://data.cityofnewyork.us/Public-Safety/Historical-New-York-City-Crime-Data/hqhv-9zeg
Philadelphia	https://www.opendataphilly.org/dataset/crime-incidents
Portland	http://www.civicapps.org/datasets
Raleigh	https://data.raleighnc.gov/Police/Police-Incident-Data-from-Jan-1-2005-Master-File/csw9-dd5k
San Francisco	https://data.sfgov.org/Public-Safety/Map-Crime-Incidents-from-1-Jan-2003/gxxq-x39z
Santa Monica	https://data.smgov.net/Public-Safety/Police-Incidents/kn6p-4y74
Seattle	https://data.seattle.gov/Public-Safety/Seattle-Police-Department-Police-Report-Incident/7ais-f98f
St. Louis	http://www.slmpd.org/Crimereports.shtml

Additionally, a `date` field that provides when a offense occurred is available in the U.S. data sets. This field may present different granularity such as the day including hour or the part of the day (e.g., morning, evening). In the case of UK data, an event does not present the date, but only the month. We did not use U.K. data for the dynamic analysis, and we limited our analysis on day level for the American cities.

Police offices may employ different terms when referring to certain types of crime. Also, they may include subcategories of a type of crime (e.g., bank robbery and gas station robbery). In order to analyze specific types crimes, we grouped together events in each city which are described by different terms but are under the same branch of a certain crime type; for that we used as a guide the definitions from FBI[2]. That is, we renamed the type of crime in each record to the broad term. Table 2, Table 3, and Table 4, contains the terms that are used by each police office and that we grouped to analyze, respectively, *theft*, *robbery*, and *burglary*. Note that some cities have only information of the broad term.

[2] https://ucr.fbi.gov/crime-in-the-u.s/2010/crime-in-the-u.s.-2010/violent-crime/violent-crime

Table 2: The terms grouped for burglary in each considered data set from U.S.

City	Terms used for burglary
Atlanta	'BURGLARY-RESIDENCE', 'BURGLARY-NONRES'
Baltimore	'BURGLARY'
Baton Rouge	'NON-RESIDENTIAL BURGLARY', 'RESIDENTIAL BURGLARY'
Boston	'Commercial Burglary', 'Residential Burglary', 'Other Burglary', 'RESIDENTIAL BURGLARY', 'COMMERCIAL BURGLARY'
Chattanooga	'BURGLARY / BREAKING AND ENTERING', 'BURGLARY', 'residential burglary', 'BURGLARY / BREAKING AND ENTERING', 'Burglary', 'Possession of Burglary Tools', 'burglary', 'BURGLARY / BREAKING AND ENTERING, Residential', 'Residential Burglary'
Chicago	'BURGLARY'
Dallas	'BURGLARY-RESIDENCE', 'BURGLARY-BUSINESS'
Denver	'burglary-business-no-force', 'burglary-residence-by-force', 'burglary-safe', 'burglary-vending-machine', 'burglary-residence-no-force', 'burglary-poss-of-tools', 'burglary-business-by-force'
Hartford	'05* -BURGLARY'
Kansas City	'burglary-res', 'burglary residence', 'Burglary -Resid', 'Burglary -Non Resid', 'BURGLARY NON RES', 'Burglary -Residence', 'burglary res', 'burglary', 'res burglary'
Los Angeles	'BURGLARY', 'BURGLARY, ATTEMPTED'
New York	'BURGLARY'
Philadelphia	'Burglary Non-Residential', 'Burglary Residential'
Portland	'Burglary'
Raleigh	'BURGLARY / UNLAWFUL / RES / UNK', 'BURGLARY / FORCIBLE / COMM / DAY', 'BURGLARY / FORCIBLE / RES / UNK', 'BURGLARY / UNLAWFUL / COMM / DAY', 'BURGLARY / ATT / RES / DAY', 'BURGLARY / FORCIBLE / RES / DAY', 'BURGLARY / ATT / COMM / NIGHT', 'BURGLARY / UNLAWFUL / COMM / UNK', 'BURGLARY / UNLAWFUL / RES / DAY', 'BURGLARY / UNLAWFUL / COMM / NIGHT', 'BURGLARY / ATT / COMM / DAY', 'BURGLARY / ATT / COMM / UNK', 'BURGLARY / FORCIBLE / RES / NIGHT', 'BURGLARY / ATT / RES / NIGHT', 'Burglary / Commercial or Non-Residential', 'BURGLARY / FORCIBLE / COMM / NIGHT', 'BURGLARY / UNLAWFUL / RES / NIGHT', 'BURGLARY / FORCIBLE / COMM / UNK', 'Burglary / Residential', 'BURGLARY / ATT / RES / UNK', 'ALL OTHER / POSSESSION OF BURGLARY TOOLS'
San Francisco	'BURGLARY'
Santa Monica	'Burglary-Force Non-Resd', 'Burglary -General', 'Burglary Attempt -Resd', 'Possess Burglary Tool', 'Burglary Attempt -Non-Resd', 'Burglary-Force Resd'
Seattle	'BURGLARY-SECURED PKNG CARPROWL', 'BURGLARY-FORCE-NONRES', 'BURGLARY-FORCE-RES', 'BURGLARY-NOFORCE-RES', 'BURGLARY-OTHER', 'BURGLARY-NOFORCE-NONRES', 'BURGLARY-SECURE PARKING-NONRES', 'BURGLARY-SECURE PARKING-RES'
St. Louis	'BURGLARY-OTHR NONRES / UNK TIM / UNLAW ENT / OCCUPIE', 'BURGLARY-OTHR NONRES / NIT / UNLAW ENT / UNOCCUPIED', 'BURGLARY-RESDNCE / UNK TIM / ATT FORCE ENTRY', 'BURGLARY-RESDNCE / UNK TIM / UNLW EN / OCCUPIED', 'BURGLARY-OTHR NONRES / NIT / ATT FORCIBLE ENTRY', 'BURGLARY-BUSINESS / UNK TIME / FORC ENT / UNOCCUPIED', 'BURGLARY-RESIDENCE / NIT / ATT FORCE ENTRY', 'BURGLARY-OTHR NONRES / NIT / UNLAW ENT / OCCUPIED', 'BURGLARY-RESIDENCE / DAY / FORCE ENT / UNOCCUPIED', 'BURGLARY-BUSINESS / NIT / UNLAW ENT / UNOCCUPIED','BURGLARY-BUSINESS / DAY / UNLAW ENT / UNOCCUPIED', 'BURGLARY-BUSINESS / NIT / FORCE ENT / UNOCCUPIE', 'BURGLARY-BUSINESS / UNK TIME / FORC ENT / OCCUPIED', 'BURGLARY-BUSINESS / NIT / ATT FORCIBLE ENTRY', 'BURGLARY-RESIDENCE / DAY / UNLAW ENT / OCCUPIED', 'BURGLARY-RESIDENCE / NIT / UNLAW ENT / OCCUPIED', 'BURGLARY-RESIDENCE / DAY / UNLAW ENT / UNOCCUPIED', 'BURGLARY-BUSINESS / NIT / UNLAW ENT / OCCUPIED', 'BURGLARY-RESDNCE / UNK TIM / FORC ENT / OCCUPIED', 'BURGLARY-BUSINESS / UNK TIME / UNLAW ENT / OCCUPIED', 'BURGLARY-OTHR NONRES / UNK TIM / UNLAW ENT / UNOCCUP', 'BURGLARY-OTHR NONRES / DAY / UNLAW ENT / OCCUPIED', 'BURGLARY-OTHR NONRES / UNK TIM / FORC ENT / UNOCCUPI', 'BURGLARY-RESIDENCE / NIT / FORCE ENT / OCCUPIED', 'BURGLARY-OTHR NONRES / UNK TIM / FORC ENT / OCCUPIED', 'BURGLARY-BUSINESS / UNK TIME / UNLAW ENT / UNOCCUPIE', 'BURGLARY-RESIDENCE / DAY / FORCE ENT / OCCUPIED', 'BURGLARY-OTHR NONRES / DAY / UNLAW ENT / UNOCCUPIED', 'BURGLARY-RESIDENCE / NIT / FORCE ENT / UNOCCUPIED'

	'BURGLARY-BUSINESS / DAY / UNLAW ENT / OCCUPIED', 'BURGLARY-OTHR NONRES / UNK TIM / ATT FORCIBLE ENTR', 'BURGLARY-BUSINESS / NIT / FORCE ENT / OCCUPIED', 'BURGLARY-BUSINESS / DAY / FORCE ENT / OCCUPIED', 'BURGLARY-RESDNCE / UNK TIM / FORC ENT / UNOCCUPIED', 'BURGLARY-OTHR NONRES / DAY / FORC ENT / OCCUPIED', 'BURGLARY-BUSINESS / DAY / FORCE ENT / UNOCCUPIED', 'BURGLARY-RESIDENCE / NIT / UNLAW ENT / UNOCCUPIED', 'BURGLARY-OTHR NONRES / DAY / ATT FORCIBLE ENTRY', 'BURGLARY-OTHR NONRES / NIT / FORC ENT / OCCUPIED', 'BURGLARY-RESIDENCE / DAY / ATT FORCE ENTRY', 'BURGLARY-OTHR NONRES / NIT / FORC ENT / UNOCCUPIED', 'BURGLARY-RESDNCE / UNK TIM / UNLW EN / UNOCCUPIED', 'BURGLARY-BUSINESS / DAY / ATTEMPT FORCIBLE ENTRY', 'BURGLARY-OTHR NONRES / DAY / FORC ENT / UNOCCUPIED'

Table 3: The terms grouped for robbery in each considered data set from U.S.

City	Terms used for burglary
Atlanta	'ROBBERY-RESIDENCE', 'ROBBERY-COMMERCIAL', 'ROBBERY-PEDESTRIAN'
Baltimore	'ROBBERY -STREET', 'ROBBERY -CARJACKING', 'ROBBERY -COMMERCIAL', 'ROBBERY -RESIDENCE'
Baton Rouge	'INDIVIDUAL ROBBERY', 'BUSINESS ROBBERY'
Boston	'ROBBERY', 'Robbery'
Chattanooga	'ROBBERY'
Chicago	'ROBBERY'
Dallas	'ROBBERY-BUSINESS', 'ROBBERY-INDIVIDUAL'
Denver	'robbery-business', 'robbery-car-jacking', 'robbery-purse-snatch-w-force', 'robbery-bank', 'robbery-residence', 'robbery-street'
Hartford	'03* -ROBBERY'
Kansas City	'Strong Arm Robbery', 'Armed Robbery'
Los Angeles	'ATTEMPTED ROBBERY', 'ROBBERY'
New York	'ROBBERY'
Philadelphia	'Robbery Firearm', 'Robbery No Firearm'
Portland	'Robbery'
Raleigh	'ROBBERY W / OTHER WEAPON -GAS STATION', 'ROBBERY / STRONGARM -CONVENIENCE STORE (PERSON)', 'ROBBERY / STRONGARM -COMMERICAL-PERSON', 'ROBBERY W / OTHER WEAPON -BANK', 'ROBBERY W / OTHER WEAPON -CONVENIENCE STORE', 'ROBBERY W / KNIFE -COMMERCIAL HOUSE', 'ROBBERY W / KNIFE -CONVENIENCE STORE-PERSON', 'ROBBERY / STRONGARM -GAS OR SVC STATION', 'ROBBERY W / OTHER WEAPON -HIGHWAY', 'ROBBERY W / KNIFE -COMMERICAL HOUSE-PERSON', 'ROBBERY / STRONGARM -MISC-PERSON', 'ROBBERY W / FIREARM -CONVENIENCE STORE', 'ROBBERY W / FIREARM -BANK -PERSON', 'ROBBERY W / OTHER WEAPON -MISC', 'ROBBERY / STRONGARM -HIGHWAY', 'ROBBERY W / KNIFE -RESIDENCE (ANYWHERE ON PREMISE)', 'ROBBERY W / OTHER WEAPON -COMMERCIAL HOUSE', 'ROBBERY / STRONGARM -COMMERCIAL HOUSE', 'ROBBERY / STRONGARM -HIGHWAY-PERSON', 'ROBBERY W / KNIFE -GAS OR SVC STATION-PERSON', 'ROBBERY W / FIREARM -HIGHWAY (STREETS,ALLEYS,ETC)', 'ROBBERY W / FIREARM -HIGHWAY -PERSON', 'ROBBERY W / KNIFE -HIGHWAY-PERSON', 'ROBBERY / STRONGARM -GAS OR SVC STATION-PERSON', 'ROBBERY W / OTHER WEAPON -COMMERICAL HOUSE-PERSON', 'ROBBERY / STRONGARM -MISC', 'ROBBERY W / KNIFE -MISC-PERSON', 'ROBBERY / STRONGARM -BANK', 'ROBBERY W / FIREARM -COMMERCIAL HOUSE', 'ROBBERY W / KNIFE -GAS OR SVC STATION', 'ROBBERY W / OTHER WEAPON -HIGHWAY-PERSON', 'Robbery / From Person', 'ROBBERY / STRONGARM -RESIDENCE', 'Robbery / From Business', 'ROBBERY W / FIREARM -MISC -PERSON', 'ROBBERY W / KNIFE -BANK', 'ROBBERY W / KNIFE -HIGHWAY (STREETS, ALLEYS, ETC)', 'ROBBERY W / FIREARM -GAS OR SERVICE STATION', 'ROBBERY W / KNIFE -MISC', 'ROBBERY W / FIREARM -BANK', 'ROBBERY / STRONGARM -RESIDENCE -PERSON', 'ROBBERY W / FIREARM -RESIDENCE (ANYWHERE ON PREMISE)', 'ROBBERY W / FIREARM -MISC', 'ROBBERY W / FIREARM -COMMERCIAL HOUSE -PERSON', 'ROBBERY W / OTHER WEAPON -BANK-PERSON', 'ROBBERY W / FIREARM -CONVENIENCE STORE -PERSON', 'ROBBERY W / KNIFE -RESIDENCE-PERSON', 'ROBBERY W / OTHER WEAPON -RESIDENCE', 'ROBBERY W / OTHER WEAPON -MISC-PERSON', 'ROBBERY W / OTHER WEAPON -RESIDENCE-PERSON', 'ROBBERY W / KNIFE -BANK-PERSON', 'ROBBERY W / OTHER WEAPON -GAS STATION-PERSON', 'ROBBERY W / FIREARM -GAS OR SVC STATION -PERSON', 'ROBBERY / STRONGARM -CONVENIENCE STORE'

	‘ROBBERY W / FIREARM -RESIDENCE -PERSON’, ‘ROBBERY / STRONGARM -BANK -PERSON’, ‘ROBBERY W / KNIFE -CONVENIENCE STORE’, ‘ROBBERY W / OTHER WEAPON -CONVENIENCE STORE-PERSON’
San Francisco	‘ROBBERY’
Santa Monica	‘Robbery-Firearm-Other Loc’, ‘Robbery Strongarm-Residential’, ‘Robbery-Firearm-Commercial’, ‘Robbery-Firearm-Residential’, ‘Robbery-Strongarm Store’, ‘Robbery-Othr Wpn-Residential’, ‘Robbery-Knife-Street / Hwy’, ‘Robbery-Knife-Other Loc’, ‘Robbery Othr Wpn-Bank’, ‘Robbery-Othr Wpn-Gas Station’, ‘Robbery-Firearm-Street / Hwy’, ‘Robbery Strongarm-Street / Hwy’, ‘Robbery-Strongarm-Commercial’, ‘Robbery-Othr Wpn-Commercial’, ‘Robbery -General’, ‘Robbery-Strongarm-Other Loc’, ‘Robbery-Othr Wpn-Other Loc’, ‘Robbery-Firearm-Gas Station’, ‘Robbery-Knife-Residential’, ‘Robbery-Knife-Bank’, ‘Robbery-Knife-Conv Store’, ‘Robbery-Strongarm-Gas Station’, ‘Robbery Strongarm-Bank’, ‘Robbery-Othr Wpn Conv Store’, ‘Robbery-Firearm-Bank’, ‘Robbery-Othr Wpn-Street / Hwy’, ‘Robbery-Firearm Conv Store’, ‘Robbery-Knife-Commercial’
Seattle	‘ROBBERY-BANK-GUN’, ‘ROBBERY-BANK-WEAPON’, ‘ROBBERY-RESIDENCE-BODYFORCE’, ‘ROBBERY-BUSINESS-BODYFORCE’, ‘ROBBERY-STREET-WEAPON’, ‘ROBBERY-RESIDENCE-WEAPON’, ‘ROBBERY-STREET-GUN’, ‘ROBBERY-STREET-BODYFORCE’, ‘ROBBERY-BANK-BODYFORCE’, ‘ROBBERY-BUSINESS-GUN’, ‘ROBBERY-RESIDENCE-GUN’, ‘ROBBERY-BANK-OTHER’, ‘ROBBERY-BUSINESS-WEAPON’, ‘ROBBERY-OTHER’
St. Louis	‘ROBBERY-COMMERCE PL / STRNGARM / INJURY / SUCCESS’, ‘ROBBERY-CONVEN STOR / STRNGARM / NO INJ / SUCCESS’, ‘ROBBERY-GAS STA / KNIFE USED / SUCCESSFUL’, ‘ROBBERY-CONVEN STOR / OTHR WEP USED / SUCCESS’, ‘ROBBERY-HIGHWAY / STRNGARM / INJURY / ATTEMPT’, ‘ROBBERY-COMMERCE PL / STRNGARM / NO INJ / SUCCESS’, ‘ROBBERY-CONVEN STOR / FIREARM USED / ATTEMPT’, ‘ROBBERY-RESIDENCE / STRNGARM / NO INJ / ATTEMPT’, ‘ROBBERY-HIGHWAY / STRNGARM / NO INJ / SUCCESS’, ‘ROBBERY-COMMERCE PL / OTHR WEP USED / SUCCESS’, ‘ROBBERY-BANK / STRNGARM / INJURY / SUCCESS’, ‘ROBBERY-HIGHWAY / OTHR WEPN USED / ATTEMPT’, ‘ROBBERY-RESIDENCE / FIREARM USED / ATTEMPT’, ‘ROBBERY-RESIDENCE / STRNGARM / INJURY / SUCCESS’, ‘ROBBERY-COMMERCE PL / KNIFE USED / SUCCESS’, ‘ROBBERY-CONVEN STOR / STRNGARM / INJURY / ATTEMPT’, ‘ROBBERY-BANK / OTHR WEPN USED / SUCCESSFUL’, ‘ROBBERY-CONVEN STOR / KNIFE USED / SUCCESS’, ‘ROBBERY-MISC / STRNGARM / NO INJ / SUCCESSFUL’, ‘ROBBERY-GAS STA / STRNGARM / NO INJ / ATTEMPT’, ‘ROBBERY-CONVEN STOR / STRNGARM / NO INJ / ATTEMPT’, ‘ROBBERY-MISC / STRNGARM / INJURY / SUCCESSFUL’, ‘ROBBERY-RESIDENCE / FIREARM USED / SUCCESSFUL’, ‘ROBBERY-CONVEN STOR / OTHR WEP USED / ATTEMPT’, ‘ROBBERY-RESIDENCE / KNIFE USED / ATTEMPT’, ‘ROBBERY-GAS STA / FIREARM USED / SUCCESSFUL’, ‘ROBBERY-GAS STA / STRNGARM / NO INJ / SUCCESS’, ‘ROBBERY-GAS STA / FIREARM USED / ATTEMPT’, ‘ROBBERY-HIGHWAY / KNIFE USED / SUCCESSFUL’, ‘ROBBERY-BANK / KNIFE USED / SUCCESSFUL’, ‘ROBBERY-COMMERCE PL / FIREARM USED / ATTEMPT’, ‘ROBBERY-BANK / FIREARM USED / SUCCESSFUL’, ‘ROBBERY-COMMERCE PL / KNIFE USED / ATTEMPT’, ‘ROBBERY-RESIDENCE / OTHR WEPN USED / SUCCESS’, ‘ROBBERY-GAS STA / STRNGARM / INJURY / ATTEMPT’, ‘ROBBERY-MISC / STRNGARM / NO INJ / ATTEMPT’, ‘ROBBERY-HIGHWAY / FIREARM USED / ATTEMPT’, ‘ROBBERY-GAS STA / STRNGARM / INJURY / SUCCESS’, ‘ROBBERY-CONVEN STOR / FIREARM USED / SUCCESS’, ‘ROBBERY-MISC / FIREARM USED / SUCCESSFUL’, ‘ROBBERY-BANK / STRNGARM / NO INJ / ATTEMPT’, ‘ROBBERY-MISC / OHTR WEO USED / SUCCESSFUL’, ‘ROBBERY-RESIDENCE / KNIFE USED / SUCCESSFUL’, ‘ROBBERY-RESIDENCE / OTHR WEPN USED / ATTEMPT’, ‘ROBBERY-HIGHWAY / KNIFE USED / ATTEMPT’, ‘ROBBERY-MISC / OTHR WEP USED / SUCCESSFUL’, ‘ROBBERY-BANK / STRNGARM / NO INJ / SUCCESS’, ‘ROBBERY-COMMERCE PL / FIREARM USED / SUCCESS’, ‘ROBBERY-BANK / FIREARM USED / ATTEMPT’, ‘ROBBERY-RESIDENCE / STRNGARM / NO INJ / SUCCESS’, ‘ROBBERY-HIGHWAY / OTHR WEPN USED / SUCCESSFUL’, ‘ROBBERY-BANK / KNIFE USED / ATTEMPT’, ‘ROBBERY-GAS STA / OTHR WEP USED / SUCCESSFUL’, ‘ROBBERY-MISC / FIREARM USED / ATTEMPT’, ‘ROBBERY-HIGHWAY / STRNGARM / NO INJ / ATTEMPT’, ‘ROBBERY-MISC / KNIFE USED / SUCCESSFUL’, ‘ROBBERY-GAS STA / OTHR WEP USED / ATTEMPT’, ‘ROBBERY-HIGHWAY / FIREARM USED / SUCCESSFUL’, ‘ROBBERY-HIGHWAY / STRNGARM / INJURY / SUCCESS’

Table 4: The terms grouped for theft in each considered data set from U.S.

City	Terms used for burglary
Atlanta	‘LARCENY-FROM VEHICLE’, ‘LARCENY-NON VEHICLE’
Baltimore	‘LARCENY’, ‘LARCENY FROM AUTO’

Baton Rouge	'THEFT'
Boston	'Larceny', 'LARCENY FROM MOTOR VEHICLE', 'OTHER LARCENY', 'Larceny From Motor Vehicle'
Chattanooga	'THEFT FROM MOTOR VEHICLE', 'THEFT FROM BUILDINGS', 'Theft of Property', 'Theft From Motor Vehicle', 'Theft from Motor Vehicle', 'Theft Under 500', 'Theft from Vehicle', 'All Other Larceny', 'Theft under $500', 'Theft', 'ALL OTHER LARCENY', 'Theft Under $500'
Chicago	'THEFT'
Dallas	'OTHER THEFTS', 'THEFT / BMV'
Denver	'theft-other', 'burg-auto-theft-busn-no-force', 'theft-from-bldg', 'burg-auto-theft-resd-no-force', 'burg-auto-theft-busn-w-force', 'burg-auto-theft-resd-w-force', 'theft-items-from-vehicle', 'theft-purse-snatch-no-force', 'theft-pick-pocket'
Hartford	'06* -LARCENY'
Kansas City	'stealing from bldg', 'Stealing from Auto', 'Stealing From Auto', 'Stealing All Other', 'Stealing from Buildi', 'Stealing Auto Parts / ', 'stealing', 'Stealing from Bldg', 'Stealing Auto Parts', 'Stealing Pickpocket', 'stealing ACC', 'stealing all other', 'Stealing From Buildi', 'stealing-bldg', 'Stealing ACC', 'stealing acc', 'Stealing other', 'stealing oth', 'STEALING', 'Stealing Purse Snatc', 'stealing from auto', 'stealing accessories', 'Stealing from buildi', 'stealing from build', 'STEALING ACC'
Los Angeles	'THEFT-GRAND ($950.01 & OVER)EXCPT,GUNS,FOWL,LIVESTK,PROD', 'THEFT FROM PERSON -ATTEMPT', 'THEFT PLAIN -PETTY ($950 & UNDER)', 'THEFT PLAIN -ATTEMPT', 'THEFT FROM MOTOR VEHICLE -ATTEMPT', 'THEFT PLAIN -PETTY (UNDER $400)', 'THEFT FROM MOTOR VEHICLE -GRAND ($400 AND OVER)', 'THEFT-GRAND (OVER $400 OR $100 IF FOWL)', 'THEFT FROM MOTOR VEHICLE -PETTY (UNDER $400)', 'BURGLARY FROM VEHICLE, ATTEMPTED', 'BURGLARY FROM VEHICLE', 'THEFT FROM MOTOR VEHICLE -PETTY ($950.01 & OVER)', 'THEFT, PERSON'
New York	'GRAND LARCENY'
Philadelphia	'Thefts', 'Theft from Vehicle'
Portland	'Larceny'
Raleigh	'LARCENY (CIVILIAN USE ONLY)', 'LARCENY / FROM BUILDING (-$50)', 'LARCENY / POCKET-PICKETING / FELONY (-$50)', 'LARCENY / PURSE-SNATCHING / FELONY (-$50)', 'LARCENY / FROM MOTOR VEHICLE / FELONY (-$50)', 'LARCENY / PURSE-SNATCHING / FELONY ($50-$199)', 'LARCENY / FROM BUILDING ($50-$199)', 'LARCENY / PURSE-SNATCHING (-$50)', 'LARCENY / POCKET-PICKING ($200-$1,000)', 'Larceny / Pocket-Picking', 'LARCENY / ALL OTHERS ($200-$1000)', 'LARCENY / ALL OTHERS ($1000+)', 'LARCENY / FROM BUILDING / FELONY ($50-$199)', 'Larceny / Theft from Building', 'Larceny / All Other', 'LARCENY / MOTOR VEHICLE PARTS / ACC / FELONY($200-1000)', 'Larceny / Purse-Snatching', 'LARCENY / FROM MOTOR VEHICLES / FELONY (OVER $1,000)', 'LARCENY / ALL OTHERS ($50-$199)', 'LARCENY / PURSE-SNATCHING / FELONY ($200-1,000)', 'LARCENY / MOTOR VEHICLE PARTS / ACC ($200-$1,000)', 'Larceny / Theft from Motor Vehicle', 'LARCENY / FROM MOTOR VEHICLES ($50-$199)', 'LARCENY / PURSE-SNATCHING ($50-$199)', 'LARCENY / FROM BUILDING / FELONY (OVER $1,000)', 'LARCENY (NO LONGER USED)', 'LARCENY / PURSE-SNATCHING ($200-$1,000)', 'LARCENY / FROM BUILDING / FELONY (-$50)', 'LARCENY FROM MOTOR VEH (NO LONGER USED)', 'LARCENY / MOTOR VEHICLE PARTS / ACC / FELONY (OVER $1,000)', 'LARCENY / FROM MOTOR VEHICLES / FELONY ($200-$1,000)', 'LARCENY / MOTOR VEHICLE PARTS / ACC / FELONY (-$50)', 'LARCENY / MOTOR VEHICLE PARTS / ACC ($50-$199)', 'LARCENY / POCKET-PICKING / FELONY ($200-$1,000)', 'LARCENY / ALL OTHERS / FELONY(-$50)', 'LARCENY / ALL OTHERS (-$50)', 'LARCENY / PURSE-SNATCHING / FELONY (OVER $1,000)', 'LARCENY / POCKET-PICKING (-$50)', 'LARCENY / POCKET-PICKING ($50-$199)', 'LARCENY / FROM MOTOR VEHICLES ($200-$1,000)', 'LARCENY / FROM MOTOR VEHICLES (-$50)', 'LARCENY / FROM BUILDING / FELONY ($200-$1,000)', 'LARCENY / MOTOR VEHICLE PARTS / ACC / FELONY ($50-$199)', 'LARCENY / MOTOR VEHICLE PARTS / ACC (-$50)', 'LARCENY / FROM MOTOR VEHICLES / FELONY ($50-199)', 'LARCENY / POCKET-PICKING / FELONY (OVER $1,000)', 'LARCENY / POCKET-PICKING / FELONY ($50-$199)', 'LARCENY / FROM BUILDING ($200-$1,000)', 'LARCENY / ALL OTHERS / FELONY($50-$199)', 'Larceny / Theft of MV Parts-Accessories', 'LARCENY / ALL OTHERS / FELONY($200-$1000)'
San Francisco	'LARCENY / THEFT'
Santa Monica	'Larceny -Purse-snatch', 'Larceny -Pickpocket', 'Larceny -General', 'Larceny -Vehicle Parts / Acc', 'Larceny -From Building', 'Larceny -Other', 'Larceny -From Vehicle'

Seattle	'THEFT-AUTO PARTS', 'THEFT-PRSNATCH', 'THEFT-OTH', 'THEFT-CARPROWL', 'THEFT-BUILDING', 'THEFT-PKPOCKET', 'THEFT-BICYCLE', 'THEFT-AUTOACC'
St. Louis	'LARCENY-PICKPOCKET $500-$24,999', 'LARCENY-MTR VEH PARTS OVER $25,000', 'LARCENY-FROM MTR VEH OVER $25,000', 'LARCENY-ALL OTHER UNDER $500 / ATTEMPT', 'LARCENY-PURSESNATCH UNDER $500 / ATTEMPT', 'LARCENY-FROM BUILDING $500 -$24,999 / ATTEMPT', 'LARCENY-ALL OTH / FRM PRSN / $150-$199.99', 'LARCENY-ALL OTHER $500 -$24,999', 'LARCENY-FROM MTR VEH $500 -$24,999', 'LARCENY-FROM MTR VEH UNDER $500 / ATTEMPT', 'LARCENY-MTR VEH PARTS $500 -$24,999', 'LARCENY-PICKPOCKET UNDER $500 / ATTEMPT', 'LARCENY-FROM BUILDING UNDER $500 / ATTEMPT', 'LARCENY-MTR VEH PARTS UNDER $500 / ATTEMPT', 'LARCENY-PURSESNATCH UNDER $500', 'LARCENY-ALL OTH / FRM PRSN / UNDER $500', 'LARCENY-FROM BLDG $200-$749.99', 'LARCENY-ALL OTHER OVER $25,000', 'LARCENY-PICKPOCKET UNDER $500', 'LARCENY-ALL OTHER / $150-$199.99', 'LARCENY-FROM MTR VEH UNDER $500', 'LARCENY-PURSESNATCH $500-$24,999', 'LARCENY-FROM BUILDING $500 -$24,999', 'LARCENY-ALL OTHER UNDER $500', 'LARCENY-FROM BUILDING UNDER $500', 'LARCENY-FROM BUILDING OVER $25,000', 'LARCENY-MTR VEH PARTS UNDER $500', 'LARCENY-FROM BLDG $150-$199.99', 'LARCENY-PICKPOCKET OVER $25,000'

Police forces in U.K. employ the same terms for crime regardless of region. Therefore, we grouped the categories of offenses in UK the same way for all considered regions, as described in Table 5.

Table 5: The terms grouped each type of crime in the data set from U.K.

Crime	Terms used
Theft	'Theft from the person', 'Other theft'
Burglary	'Burglary'
Robbery	'Robbery'

1.2 Geospatial information

In the case of the U.S. cities, we obtained the boundaries of the U.S. states from the U.S. Census Bureau[3]. We used the TIGER (Topologically Integrated Geographic Encoding and Referencing) shapefiles with granularity of blocks (delimited in 2010). To have only the regions of the considered cities in the study, we clipped each shapefile with the bounding box of each city. The bounding boxes were retrieved from the OpenStreetMap initiative[4]. For the U.K. data, we gathered the boundaries of the jurisdiction of each police forces (from December 2011)[5], then clipped them with the boundaries (super generalized clipped boundaries in England and Wales) of the Lower Layer Super Output Areas (LSOAs)[6].

In order to carry out spatial analyses, we have to project each crime data set on the same projection of their respective boundaries. Since most of the crime data sets and boundaries have the same spatial reference (`EPSG:4326`), this procedure was only needed to be performed on few data sets, as described in Table 6.

Table 6: The spatial references in the crime data sets and the shapes of the locations.

Location	Crime data sets	Boundaries
St. Louis	EPSG:2815	EPSG:4326
Portland	EPSG:2269	EPSG:4326
Dallas	EPSG:2276	EPSG:4326
Other US Cities	EPSG:4326	EPSG:4326
UK Data	EPSG:4326	EPSG:27700

[3] https://www.census.gov/
[4] http://nominatim.openstreetmap.org/
[5] https://data.police.uk/data/boundaries/
[6] http://geoportal.statistics.gov.uk/

1.3 Population

We gathered data with respect to the total resident population in smallest spatial units available of the considered locations from official census. In the case of the U.S. cities, we used the 2010 census data from the U.S. Census Bureau[7] which provides the total population (P1) in block level. For the locations in the U.K., our analysis were done with the 2011 census data in LSOA level provided by the Office for National Statistics[8].

2 Splitting cities

To split a city in k regions with same population size, we first create a graph based on the spatial and census data in which each node of the graph represents the same amount of population. Then, we partition the graph in parts with the same number of nodes and thus the total population in each region is also the same.

Such graph is constructed based on the cells of the Voronoi diagrams derived from random coordinates uniformly generated within each shape in the city. The number of points created in each shape s_i is proportional to its resident population p_i. In Pseudocode 1, a description is given for the main steps we employ to split locations. The core of our procedure comprises mainly of three functions:

- `GenerateRandomCoordinatesOnShape(n=number of coordinates, s=shape)` — This function returns a list containing n coordinates that are randomly generated uniformly within a given shape s.
- `ClippedVoronoi(c=list of coordinates, s=shape)` — This function returns a list with the shapes of the cells from the Voronoi diagram derived from the coordinates in s after post-processing the cells by clipping them with the shape s. For our experiments, in order to create the diagrams, we employed the library `qhull`[9] which is wrapped in the `pyhull` library[10].
- `Partition(g=graph, k=number of partitions)` — This function splits a graph in k parts of the same size while minimizing the number of edges between nodes of different parts, then returns a list d with elements d_i equals to the index $j \in [1, k]$ of the partition of the node n_i. In this work, we used the KaFFPa (Karlsruhe Fast Flow Partitioner) algorithm to partition the graphs [1].

Finally, we group the shapes s from the output of `SplitLocation` based on their partition index $j \in [1, k]$. We define each of these k groups as a region r_j in the city. For the purpose of our work here, we say that a criminal event occurred in region r_j if the offense took place on a shape that belongs to the group of shapes of the region r_j.

2.1 The number of splits and the analysis of crime

To analyze crime in a given city c with the method described in Section 2, we have to choose the number of regions R that the city will be divided. This value has to be chosen in such way that the aggregation level leads to units that represent the place. The analysis of crime at the wrong geographic unit may lead to incorrect understanding of crime dynamics [2]. Such problem can arise by examining at larger spatial levels which might hide lower-order variability (i.e., the averaging problem), an 150-years-old observation documented by Glyde [3]. The focus on micro levels, on the other hand, might obscure the importance and impact of larger community and neighborhood effects [2].

In order to find a suitable split in the cities, we divided each city by an increasing number of splits and analyze the number of regions without any crime. We found that the number of regions with at

[7] `https://factfinder.census.gov/`
[8] `https://www.ons.gov.uk`
[9] `http://www.qhull.org/`
[10] `http://pythonhosted.org/pyhull/`

Pseudocode 1: Given a location L that is composed by different shapes b_i and a real-valued quantity about each b_i, split L in k regions such that the sums of the quantities in the regions are roughly equal to the same amount.

input : list of shapes b; list of real numbers p; number of regions k
output : list of shapes s; list of integers d
parameter: granularity level r (default = 1.0)

Function *SplitLocation(b, p, k, r = 1.0):*
    $s \leftarrow$ `List()`
    foreach *shape* b_i *in* b **do**
        $t_i \leftarrow$ `GenerateRandomCoordinatesOnShape(`rp_i, b_i`)`
        $v_i \leftarrow$ `ClippedVoronoi(`t_i, b_i`)`
        $s \leftarrow$ `Concatenate(`s, v_i`)`
    end
    Create a graph G with $\|s\|$ disconnected nodes
    foreach *node* n_j *in* G **do**
        foreach *node* n_k *in* G **do**
            if s_j *is spatially adjacent to* s_k **then**
                Create an edge between n_j and n_k
            end
        end
    end
    $d \leftarrow$ `Partition(`G, k`)`
    return (s, d)

least one crime $R^{n \geq 1}$ increases with the total number of regions R, until $R^{n \geq 1}$ saturates at a certain point u in such way that $R^{n \geq 1}(r') = u$ for $r' \geq r_u$ (shown in Figure 1). In other words, even if we divide a city in more regions, after this point the number of regions with crime is the same. A plausible reason for that is the accuracy level used in police departments when an offense is registered in the criminal system. An ideal data set of crime would be one that the number of regions with crime increases steadily with the number of regions, until the number of regions is equal to the number of offenses (i.e., each criminal event has its own region). However, such ideal data is difficult to exist due to aspects in the very nature of the data. Since we are working with *spatial* data, the limitations in the apparatus involved to store events, such as coordinates look-up in GIS systems or GPS receivers, may introduce inaccuracies in the data. For instance, an office could use a GIS system that provides the same coordinates of a location regardless where this location is on a street. Moreover, *criminal* data has sensible information with respect to criminals, victims, and ongoing investigations, thus police offices might intentionally decrease the granularity of the data for privacy purposes.

Nevertheless, the procedures that each office carries out to record a criminal event lead to different levels of accuracy in the data sets. Analyses using geographic units smaller than the limits found in each city have the potential to be biased by the arbitrary system in offices. In order not to bias our results with such procedures and for the purpose of our analysis, we set $R_c = \rho r_u$ with $\rho = 0.9$. The vertical lines in Figure 1 are the values for R_c regarding different types of crime. Some statistics of crime for each location after we split them are described in Table 7.

2.2 Arrangements

Each city c can be divided into R_c regions in different ways or arrangements. In fact, the numbers in Table 7 are related to a certain arrangement of divisions in each city. Since we want to analyze crime in a city and not in arbitrary arrangement of divisions, we create distinct arrangements of R_c regions for each city c. For that, we employ a stochastic partitioning algorithm with the method described in

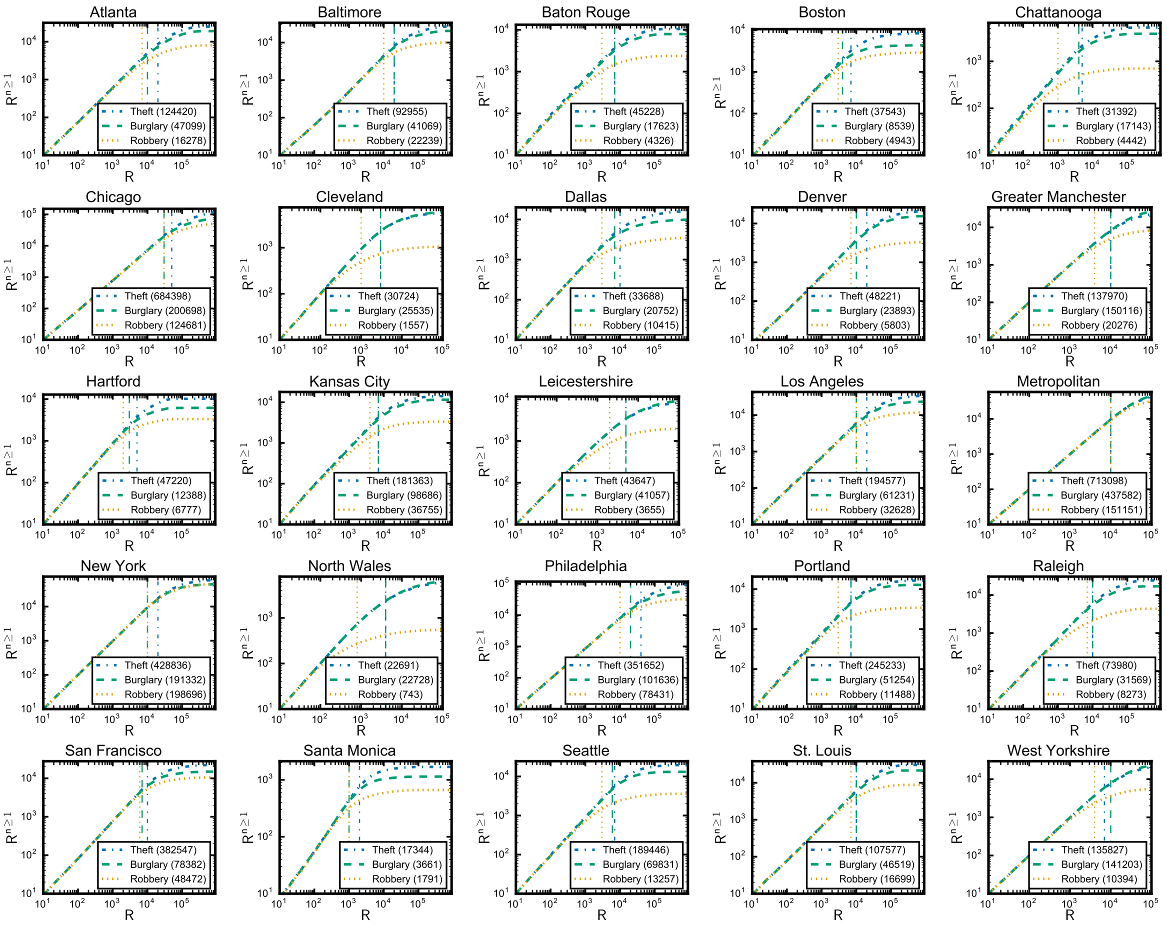

Figure 1: The number of regions that contains at least one offense $R^{n\geq 1}$ increases with the total number of regions R, until $R^{n\geq 1}$ saturates at a point $R^{n\geq 1}(r_u) = u$ in which new regions do not have any crime occurring within them. We split each city c in $R_c = \rho r_u$ regions where we set $\rho = 0.9$.

Section 2 for splitting the cities. We use the KaFFPa (Karlsruhe Fast Flow Partitioner) algorithm to partition a city 30 times using different seeds for the random number generator [1]. Hence, for each city c we first generated 30 arrangements in which each comprises of R_c same-population divisions of the city, then aggregated the occurrences of crime by type of crime such as theft, burglary, and robbery; the aggregation is done for each arrangement.

3 Model

We followed the procedures described by Clauset et al. in order to select the models for the distributions of crime in the considered locations [4]. For each empirical distribution of crime in an arrangement of a city, we follow these steps: (1) we estimate x_{min} and α of the power law; (2) calculate the goodness-of-fit of the power-law model; (3) fit the data with the following distributions: truncated power law (TP), lognormal (LN), exponential (EX), and stretched exponential (SE); and (4) compare the power-law model with the other models using the likelihood ratio test. To estimate the parameters of the power law, we employed the methods described by Clauset et al. which are implemented in the Python library `powerlaw` [4, 5]. In the case of the (4) step, we do not trust the result of the test when the

Table 7: **Criminal statistics for each location after splitting them in $R_c = \rho r_u$ regions.**

	Burglary				Robbery				Theft			
Location	n	$\bar{x}$	S	x_{max}	n	$\bar{x}$	S	x_{max}	n	$\bar{x}$	S	x_{max}
Atlanta	47099	6.33	10.33	207	16278	3.54	5.37	95	124420	14.30	61.18	3364
Baltimore	41069	5.50	5.08	113	22239	4.09	6.38	197	92955	11.39	32.16	1179
Baton Rouge	17623	5.50	7.08	100	4326	2.94	3.44	37	45228	12.40	43.15	2001
Boston	8539	3.43	3.11	45	4943	2.86	3.99	64	37543	11.62	35.19	876
Chattanooga	17143	9.14	8.00	85	4442	8.69	8.84	73	31392	14.67	26.98	657
Chicago	200698	6.84	6.83	97	124681	5.34	9.17	274	684398	20.51	73.39	3867
Cleveland	25535	11.04	11.08	106	1557	2.08	2.04	16	30724	13.43	26.04	571
Dallas	20752	4.48	6.39	156	10415	5.06	6.93	88	33688	5.84	13.02	360
Denver	23893	3.93	3.96	68	5803	2.73	4.48	109	48221	7.27	18.60	836
Greater Manchester	150116	18.00	15.56	291	20276	4.11	6.64	218	137970	17.29	59.44	2072
Hartford	12388	3.86	3.73	46	6777	3.23	4.54	78	47220	12.55	40.87	1293
Kansas City	98686	26.05	44.80	1178	36755	19.15	78.20	1633	181363	42.72	134.48	3584
Leicestershire	41057	12.04	11.51	125	3655	2.78	3.81	51	43647	13.21	37.16	1114
Los Angeles	61231	6.31	6.15	89	32628	4.96	7.89	117	194577	17.92	34.78	1714
Metropolitan	437582	45.20	28.96	512	151151	16.34	24.18	920	713098	73.69	248.35	9487
New York	191332	11.47	12.55	500	198696	12.15	15.90	407	428836	24.37	73.99	4156
North Wales	22728	9.66	11.21	114	743	1.76	1.42	11	22691	9.76	16.96	419
Philadelphia	101636	4.06	5.06	241	78431	4.28	7.22	256	351652	11.88	54.36	3597
Portland	51254	11.62	11.86	161	11488	5.55	13.62	332	245233	52.12	188.32	7137
Raleigh	31569	5.75	6.94	128	8273	3.89	6.55	92	73980	12.22	27.74	646
San Francisco	78382	12.11	27.51	861	48472	9.17	27.92	1245	382547	54.63	258.87	13431
Santa Monica	3661	5.35	5.44	47	1791	4.01	8.28	98	17344	22.18	49.48	642
Seattle	69831	13.15	14.03	252	13257	6.14	15.09	344	189446	33.15	83.31	2173
St. Louis	46519	8.13	6.86	75	16699	4.10	6.09	114	107577	17.53	36.20	1093
West Yorkshire	141203	23.77	19.49	285	10394	3.32	4.12	68	135827	23.18	77.14	2858

associated p-value is greater than 0.1. For the (2) step, we reject the power-law model in the case that the estimated p-value is lesser than 0.1. Since we have different arrangements for each city for a given type of crime, we define the score of the power law as the relative number of times that the power-law model was not rejected in a city–crime pair (see Table 8).

Furthermore, for each city, we rule out the power law to describe the distribution of crime if the score is less than 0.9. In our experiment, this case will happen if less than 27 arrangements out of 30 do not satisfy the aforementioned p-value conditions. Our results showed that the score requirement was not attained only in 1 location for thefts, 3 locations for robberies, and 1 location for burglaries, out of the 25 considered locations (see the bold scores in Table 8).

For each set of arrangements, we count the number of times each alternative distribution is (or not) statistically favored over the power law. Table 8 summarizes this counting in which l denotes the count that the alternative happens to be favored, while w represent the contrary. Note that, for each alternative model, type of crime, and city, the sum $w + l$ is not necessary equal to the number of arrangements n_a. In fact, $n_a - (w + l)$ is the number of times the results from the likelihood ratio test can not be trusted due to large p-value [4]. Here we consider that an alternative is favored in a city over the power law if $l/n_a > 2/3$ (i.e., if the alternative is favored more than 2/3 of the time). In our case, such requirement means $l \geq 21$. Still, if more than one alternative is favored over the power law in the city, we say that the one with higher l is the one favored over the others. As described in Table 8, the truncated power law was favored over the power law in 4 locations for thefts, 5 locations for robberies, and 2 locations for burglaries. In Table 9, the estimated parameters found for each city–crime pair are summarized, and Figure 2–4 depict the complementary cumulative distribution function (CCDF) for the distributions with the parameters found along with the empirical CCDF for each city.

As highlighted in the main text, the exponents of the distributions of burglary and robbery tend to present high values which means that criminal events tend to concentrate less in the case of such types of crime. The high-valued exponents must be taken with caution due to the behavior of the

Table 8: **PL: power law; LN: lognormal; EX: exponential; SE: stretched exponential; TPL: truncated power law**

	Burglary									Robbery									Theft								
	PL	LN		EX		SE		TP		PL	LN		EX		SE		TP		PL	LN		EX		SE		TP	
City	score	w	l	w	l	w	l	w	l	score	w	l	w	l	w	l	w	l	score	w	l	w	l	w	l	w	l
Atlanta	**30/30**	0	1	30	0	2	0	0	1	**30/30**	0	2	26	0	0	0	0	0	**28/30**	0	1	30	0	0	1	0	27
Baltimore	**30/30**	0	4	19	0	4	0	0	0	**29/30**	0	1	16	0	0	0	0	1	**28/30**	0	0	30	0	25	0	0	1
Baton Rouge	**30/30**	0	9	30	0	10	0	0	0	**30/30**	0	9	28	0	9	0	0	0	**30/30**	7	2	30	0	30	0	0	0
Boston	**30/30**	0	4	23	0	9	0	0	0	**30/30**	1	10	30	0	20	0	0	0	**30/30**	3	15	30	0	30	0	0	0
Chattanooga	**30/30**	0	5	26	0	0	0	0	0	**27/30**	0	2	2	0	0	3	0	21	**30/30**	0	0	30	0	0	0	0	0
Chicago	**22/30**	0	13	0	19	0	13	0	28	19/30	0	18	0	4	0	19	0	30	**30/30**	0	0	30	0	16	0	0	1
Cleveland	**28/30**	0	1	1	1	0	1	0	13	**30/30**	0	19	24	0	6	0	0	0	**30/30**	0	0	30	0	4	0	0	3
Dallas	**30/30**	0	0	25	0	0	0	0	0	**30/30**	0	4	17	0	0	0	0	2	**30/30**	0	5	30	0	7	0	0	0
Denver	**29/30**	0	16	30	0	19	0	0	0	**27/30**	0	0	7	0	0	0	0	4	**30/30**	1	0	30	0	30	0	0	0
Greater Manchester	**30/30**	0	0	11	0	0	0	0	2	**29/30**	0	2	24	0	1	0	0	0	**30/30**	4	5	30	0	30	0	0	0
Hartford	**30/30**	0	6	10	0	0	0	0	0	**29/30**	0	0	1	0	0	0	0	2	**30/30**	3	2	30	0	30	0	0	0
Kansas City	**25/30**	1	9	30	0	30	0	0	0	**30/30**	0	16	30	0	28	0	0	0	**30/30**	0	0	30	0	27	0	0	0
Leicestershire	**28/30**	0	1	2	0	0	0	0	7	**30/30**	0	16	28	0	6	0	0	0	**30/30**	1	18	30	0	30	0	0	0
Los Angeles	**30/30**	0	3	18	0	1	0	0	2	**25/30**	0	3	28	0	0	5	0	27	**30/30**	0	0	30	0	8	0	0	0
Metropolitan	**29/30**	2	7	30	0	30	0	0	0	**30/30**	0	1	30	0	23	0	0	0	**27/30**	0	0	30	0	0	0	0	26
New York	**29/30**	3	1	30	0	30	0	0	0	21/30	0	12	8	0	0	13	0	29	**29/30**	0	0	30	0	27	0	0	0
North Wales	**30/30**	0	0	27	0	0	0	0	4	**30/30**	0	8	27	0	21	0	0	0	**30/30**	0	0	30	0	0	0	0	1
Philadelphia	**30/30**	3	1	30	0	30	0	0	0	**28/30**	0	0	30	0	0	0	0	25	**30/30**	3	8	30	0	30	0	0	0
Portland	**30/30**	0	2	30	0	3	0	0	0	**30/30**	0	0	24	0	0	0	0	5	**30/30**	0	0	30	0	30	0	0	0
Raleigh	**29/30**	0	5	28	0	5	0	0	1	**30/30**	0	0	23	0	0	0	0	4	**30/30**	0	0	30	0	1	0	0	29
San Francisco	**30/30**	1	12	30	0	29	0	0	0	**28/30**	0	0	30	0	0	0	0	19	**30/30**	0	0	30	0	0	0	0	21
Santa Monica	**30/30**	0	15	29	0	6	0	0	0	**30/30**	0	2	29	0	0	0	0	0	**30/30**	0	2	30	0	7	0	0	0
Seattle	**28/30**	0	1	30	0	0	1	0	29	**28/30**	0	0	28	0	0	0	3	9	**30/30**	0	2	30	0	30	0	0	1
St. Louis	**30/30**	0	10	29	0	12	0	0	0	**29/30**	0	0	30	0	1	0	0	12	**30/30**	1	13	30	0	30	0	0	0
West Yorkshire	**28/30**	0	2	19	0	0	1	0	3	**30/30**	0	19	30	0	19	0	0	0	**30/30**	3	2	30	0	30	0	0	0

power law as the α exponent grows, as depicted in Figure 5. The concentration rapidly vanishes when α increases, and starts to present levels of concentration similar to an exponential, that is, there is almost no concentration.

4 Independence test for alpha vs population

To evaluate the relationship between the concentration of crime in a city and the population size of this city, we use a test of independence proposed by Hoeffding [6, 7]. The null hypothesis here is that two random variables X and Y are independent, that is:

$$\mathrm{H}_0: \quad F_{X,Y}(x,y) \equiv F_X(x)F_Y(y) \quad \forall\,(x,y), \tag{1}$$

where $F_{X,Y}(x,y)$ is the joint distribution of X and Y and their respective marginal distributions are $F_X(x)$ and $F_Y(y)$. For the alternative that X and Y are dependent, we reject H_0, at the α level of confidence, if $D \geq d_\alpha$ where D is the Hoeffding test statistic and d_α satisfies $P(D \geq d_\alpha) = \alpha$. Since we deal with a small sample size, we use `HoeffD` function from the `NSM3` library in `R` developed by [6]. In this analysis, we focused only on the U.S. cities in order to keep all the data points in the same urban system [8]. For that, we used the mean α over the 30 arrangements for each city–crime pair and the population size from the 2010 U.S. Census. We found $D = 0.000067$ for theft, $D = -0.001260$ for robbery, and $D = -0.000267$ for burglary. Therefore, we do not reject the null hypothesis with the 95% confidence ($d_\alpha = 0.003$).

Table 9: **Estimated parameters for the power-law distributions and the truncated power-law distribution.** Locations where the truncated power-law is favored over the power law are indicated by the λ of the distribution.

	Burglary			Robbery			Theft		
Location	x_{min}	α	λ	x_{min}	α	λ	x_{min}	α	λ
Atlanta	22	3.224 ± 0.015		21	3.516 ± 0.037		47	2.190 ± 0.016	0.0005 ± 0.00003
Baltimore	19	4.894 ± 0.068		17	3.311 ± 0.031		10	2.493 ± 0.005	
Baton Rouge	11	3.227 ± 0.019		9	3.548 ± 0.049		8	2.326 ± 0.006	
Boston	13	5.292 ± 0.109		10	3.344 ± 0.045		10	2.412 ± 0.008	
Chattanooga	28	5.059 ± 0.096		18	1.400 ± 0.145	0.0299 ± 0.00279	24	2.730 ± 0.015	
Chicago	37	4.473 ± 0.227	0.0178 ± 0.00393	31	2.404 ± 0.082	0.0100 ± 0.00086	21	2.433 ± 0.002	
Cleveland	27	3.936 ± 0.056		6	3.256 ± 0.057		21	2.744 ± 0.013	
Dallas	18	3.722 ± 0.041		21	3.504 ± 0.039		14	2.691 ± 0.013	
Denver	15	4.240 ± 0.035		13	3.096 ± 0.041		6	2.603 ± 0.006	
Greater Manchester	49	4.685 ± 0.056		21	3.312 ± 0.027		18	2.414 ± 0.006	
Hartford	17	5.119 ± 0.104		18	4.004 ± 0.068		13	2.443 ± 0.007	
Kansas City	47	3.402 ± 0.019		23	2.378 ± 0.011		41	2.349 ± 0.006	
Leicestershire	32	4.460 ± 0.062		8	2.885 ± 0.031		19	2.534 ± 0.007	
Los Angeles	30	4.864 ± 0.062		18	2.512 ± 0.074	0.0101 ± 0.00125	32	2.826 ± 0.009	
Metropolitan	78	5.321 ± 0.034		43	3.542 ± 0.019		177	2.283 ± 0.015	0.0001 ± 0.00001
New York	42	4.199 ± 0.020		57	2.681 ± 0.124	0.0086 ± 0.00092	27	2.465 ± 0.005	
North Wales	25	3.649 ± 0.039		6	3.627 ± 0.139		25	2.980 ± 0.020	
Philadelphia	20	4.306 ± 0.025		22	2.793 ± 0.043	0.0046 ± 0.00047	10	2.428 ± 0.003	
Portland	25	3.725 ± 0.028		22	2.663 ± 0.025		32	2.290 ± 0.004	
Raleigh	18	3.856 ± 0.033		13	2.905 ± 0.026		17	2.286 ± 0.016	0.0013 ± 0.00011
San Francisco	29	2.916 ± 0.015		21	2.445 ± 0.009		102	2.146 ± 0.012	0.0001 ± 0.00001
Santa Monica	12	3.606 ± 0.053		6	2.391 ± 0.022		18	2.559 ± 0.015	
Seattle	26	2.939 ± 0.061	0.0074 ± 0.00078	25	2.598 ± 0.021		23	2.373 ± 0.004	
St. Louis	24	5.951 ± 0.079		10	2.912 ± 0.019		15	2.700 ± 0.008	
West Yorkshire	49	4.952 ± 0.053		11	3.400 ± 0.030		23	2.546 ± 0.007	

5 Scaling laws of crime

We followed the method developed by Leitão et al. to evaluate the relationship $Y = \alpha N^{\beta}$ between the size of cities N and the amount of crime Y in the cities. Such method does not assume that the fluctuations around $\ln y$ and $\ln x$ are normally distributed [13]. For that, the probability $\mathrm{P}(Y|N)$ is modeled by city and person models. In the first, $\mathrm{P}(Y|N)$ is possible to be given by (*i*) Gaussian fluctuations and (*ii*) log-normal fluctuations; while the latter is a simple model which incorporates the thrust that individuals receive tokens based on the population of the city where they live (more details in [13]). We used an implementation developed by Leitão et al.[11] in order to estimate the value of β by employing each of these models. In Table 10, we summarize the β values found with respect to different types of crime from U.S. cities as provided by the Federal Bureau of Investigations (FBI), specifically the "Offenses Known to Law Enforcement, by State by City, 2015" table[12]. Figure 6 depicts the scaling of crime in the case of burglary, robbery, and theft.

In the same spirit as described by Leitão et al, for each model, we calculate ΔBIC that is the difference between the Bayesian Information Criteria (BIC) of the model and the same model with fixed $\beta = 1$ [13]. The rationale here is to test the model against a linear model and three outcomes may occur: (1) if $\Delta BIC < 0$, we say that the model is linear ($\longrightarrow$), (2) in the case of $\Delta BIC > 6$, the model is super-linear ($\nearrow$) or sub-linear ($\searrow$), and (3) if $0 < \Delta BIC < 6$, the model is inconclusive $\circ$.

[11] `https://github.com/edugalt/scaling/`

[12] `https://ucr.fbi.gov/crime-in-the-u.s/2015/crime-in-the-u.s.-2015`

Table 10: **Estimated parameters for the scaling laws of different between the size of the cities and the amount of crime.** The data is from U.S. which is provided by the Federal Bureau of Investigations (FBI). Bold represents the lowest value of BIC found. In all cases $p < 0.05$.

	City model				
	Log-normal		Gaussian		
Crime type	$\delta = 2$	$\delta \in [1, 3]$	$\delta = 1$	$\delta \in [1, 2]$	Person model
Auto theft	**0.96** (0.02) $\searrow$	0.97 (0.03) $\circ$	1.15 (0.10) $\nearrow$	1.15 (0.30) $\longrightarrow$	1.15 (0.14) $\nearrow$
Agg. assault	**0.86** (0.02) $\searrow$	0.85 (0.02) $\searrow$	1.08 (0.12) $\longrightarrow$	1.28 (0.08) $\nearrow$	1.14 (0.05) $\nearrow$
Arson	**0.49** (0.03) $\searrow$	0.57 (0.04) $\searrow$	0.67 (0.22) $\searrow$	1.14 (0.11) $\nearrow$	1.02 (0.05) $\longrightarrow$
Burglary	0.98 (0.01) $\circ$	0.98 (0.01) $\longrightarrow$	1.07 (0.06) $\nearrow$	1.17 (0.17) $\longrightarrow$	1.05 (0.09) $\longrightarrow$
Larceny–theft	**1.06** (0.01) $\nearrow$	1.13 (0.01) $\nearrow$	1.05 (0.04) $\nearrow$	1.08 (0.07) $\nearrow$	1.03 (0.05) $\longrightarrow$
Murder	**0.43** (0.05) $\searrow$	0.55 (0.05) $\searrow$	0.68 (0.10) $\searrow$	1.11 (0.12) $\longrightarrow$	0.95 (0.12) $\longrightarrow$
Property crime	**1.07** (0.01) $\nearrow$	1.12 (0.01) $\nearrow$	1.07 (0.05) $\nearrow$	1.10 (0.07) $\nearrow$	1.05 (0.07) $\longrightarrow$
Rape (legacy)	**0.59** (0.07) $\searrow$	0.64 (0.08) $\searrow$	0.97 (0.29) $\longrightarrow$	0.97 (0.19) $\longrightarrow$	0.97 (0.13) $\circ$
Rape (revised)	**0.66** (0.02) $\searrow$	0.71 (0.02) $\searrow$	0.76 (0.04) $\searrow$	1.08 (0.05) $\nearrow$	1.00 (0.06) $\longrightarrow$
Robbery	**0.92** (0.03) $\searrow$	0.94 (0.03) $\searrow$	1.25 (0.09) $\nearrow$	1.33 (0.21) $\nearrow$	1.24 (0.12) $\nearrow$
Violent crime	**0.96** (0.02) $\searrow$	0.95 (0.02) $\searrow$	1.17 (0.10) $\nearrow$	1.32 (0.08) $\nearrow$	1.18 (0.07) $\nearrow$

6 Entropy of rank

To analyze the dynamics of crime, we first split the data based on quantity (amount-based) and on time (time-based), then we created ranks of criminal regions (i.e., we ordered the regions based on the number of crime) for each split of data, and then calculated the entropy of the distribution of each position in the rank. The procedure to calculate the entropy H_r^c of the rank r of the city c is described in Figure 7, specifically for the time-based rank r_t; the r_a case (amount-based) presents analogous procedure.

For the purposes of our analysis, we split the data by $t_w = 7$ days in the r_t case and by $a_w = a_w^c$ records for amount-based case r_a, where a_w^c is the split size (e.g., every 50 criminal records, or every 25 records) in which the entropy is the lowest for each city c. To find a_w^c, firstly we measured the entropy of the first position, that is, $H_a^c(1)$, while increasing a_w for each arrangement of R_c split for each location c, secondly we used Tukey's test to find the set $s_{a_w}^c$ containing the values of a_w in which the mean of $H_a^c(1)$ is statistically equal to the lowest (with 95% confidence), then finally we define $a_w^c = \min(s_{a_w}^c)$ (Fig 4A of the main text). Figure 8 depicts the influence of the different size a_w on the measured entropy of the rank r_a with respect to thefts for all considered cities.

6.1 Clustering cities

To create a hierarchy of cities based on their dynamics with respect to criminal events across the regions in the cities, we clustered the entropies of the ranks using agglomerative hierarchical clustering technique which uses average as the method to calculate the distance between clusters, known as UPGMA (Unweighted Pair Group Method with Arithmetic Mean) algorithm. We chose the Euclidean distance as the metric to measure the distance between clusters. Here we used the entropy of the positions in the rank as the feature vector. For the clustering, we focused on the r_t rank and only used positions $i > 20$, since the values stabilizes over the positions. In order not to bias towards the high entropy values, we normalized the feature vector as the following:

$$x_c = [\tilde{H}_{r_t}^c(1), \tilde{H}_{r_t}^c(2), \ldots, \tilde{H}_{r_t}^c(20)], \tag{2}$$

where

$$\tilde{H}^{c}_{r_t}(k) = \frac{H^{c}_{r_t}(k)}{\sum_{c_i}^{N} H^{c_i}(k)/N}, \tag{3}$$

that is, we normalized each position k by the sample average of the entropy of the position k among all considered cities. To perform such analysis, we used the implementation of the algorithm available in the `scipy` library[13] for Python.

References

[1] Peter Sanders and Christian Schulz. Think Locally, Act Globally: Highly Balanced Graph Partitioning. In *Proceedings of the 12th International Symposium on Experimental Algorithms (SEA'13)*, volume 7933 of *LNCS*, pages 164–175. Springer, 2013.

[2] David Weisburd, Gerben JN Bruinsma, and Wim Bernasco. Units of analysis in geographic criminology: historical development, critical issues, and open questions. In *Putting crime in its place*, pages 3–31. Springer, 2009.

[3] John Glyde. Localities of crime in suffolk. *Journal of the Statistical Society of London*, pages 102–106, 1856.

[4] Aaron Clauset, Cosma Rohilla Shalizi, and M. E. J. Newman. Power-Law Distributions in Empirical Data. *SIAM Review*, 51(4):661–703, nov 2009.

[5] Jeff Alstott, Ed Bullmore, and Dietmar Plenz. powerlaw: a python package for analysis of heavy-tailed distributions. *PloS one*, 9(1):e85777, 2014.

[6] Eric Chicken Myles Hollander, Douglas A. Wolfe. *Nonparametric Statistical Methods.* Wiley Series in Probability and Statistics. John Wiley & Sons, 3rd edition, 2014.

[7] Wassily Hoeffding. A non-parametric test of independence. *The annals of mathematical statistics*, pages 546–557, 1948.

[8] Luís MA Bettencourt and José Lobo. Urban scaling in europe. *Journal of The Royal Society Interface*, 13(116):20160005, 2016.

[9] Luís M A Bettencourt, José Lobo, Dirk Helbing, C. Kuhnert, and Geoffrey B West. Growth, innovation, scaling, and the pace of life in cities. *Proceedings of the National Academy of Sciences*, 104(17):7301–7306, apr 2007.

[10] Luiz G.A. Alves, Haroldo V. Ribeiro, and Renio S. Mendes. Scaling laws in the dynamics of crime growth rate. *Physica A: Statistical Mechanics and its Applications*, 392(11):2672–2679, jun 2013.

[11] Rémi Louf and Marc Barthelemy. Scaling: Lost in the Smog. *Environment and Planning B: Planning and Design*, 41(5):767–769, oct 2014.

[12] J. C. Leitão, J. M. Miotto, M. Gerlach, and E. G. Altmann. Is this scaling nonlinear? *Royal Society Open Science*, 3(7):150649, jul 2016.

[13] Jorge C Leitão, José María Miotto, Martin Gerlach, and Eduardo G Altmann. Is this scaling nonlinear? *Royal Society Open Science*, 3(7):150649, 2016.

[13] `https://www.scipy.org/`

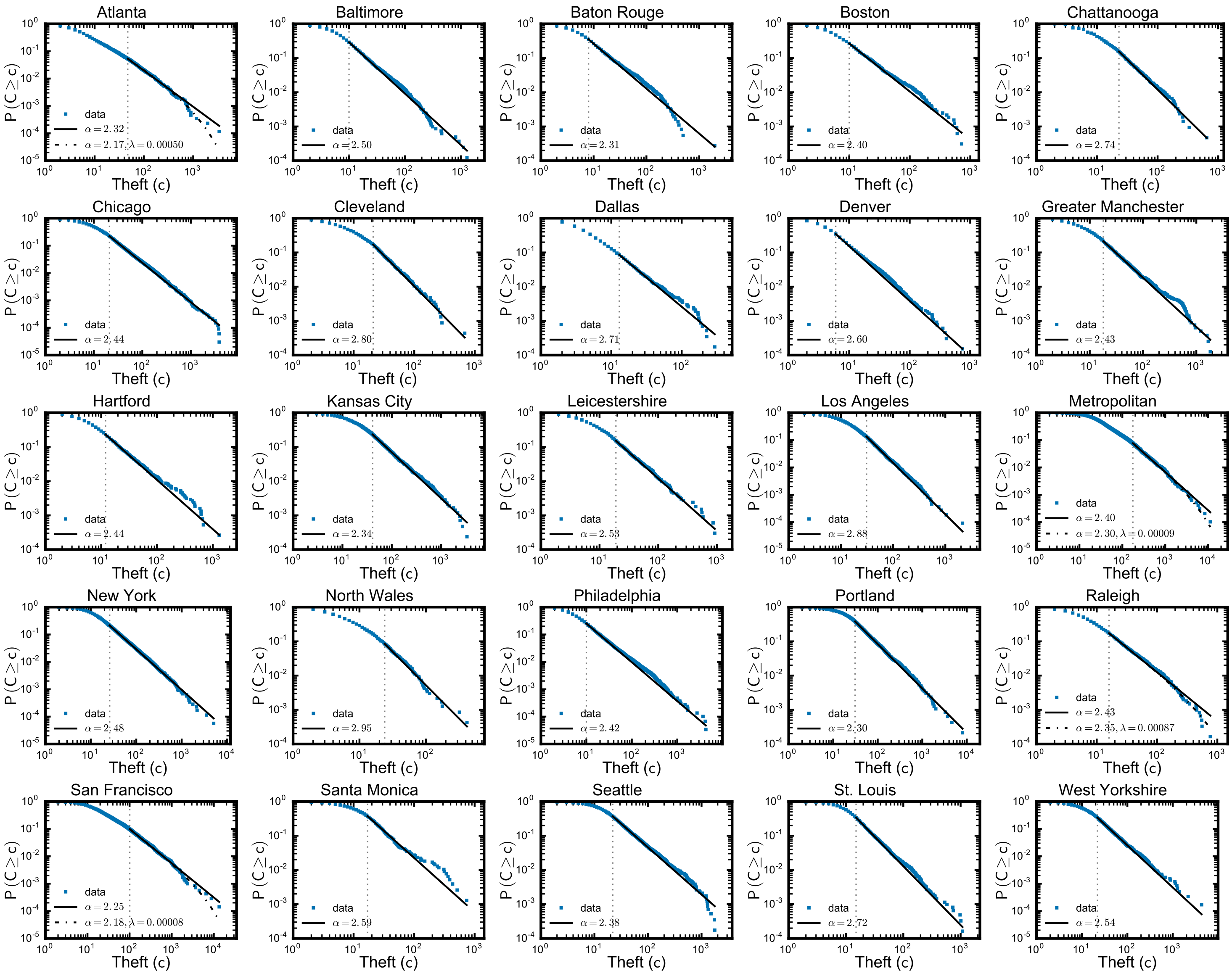

Figure 2: The best fits for the concentration of thefts in all considered cities.

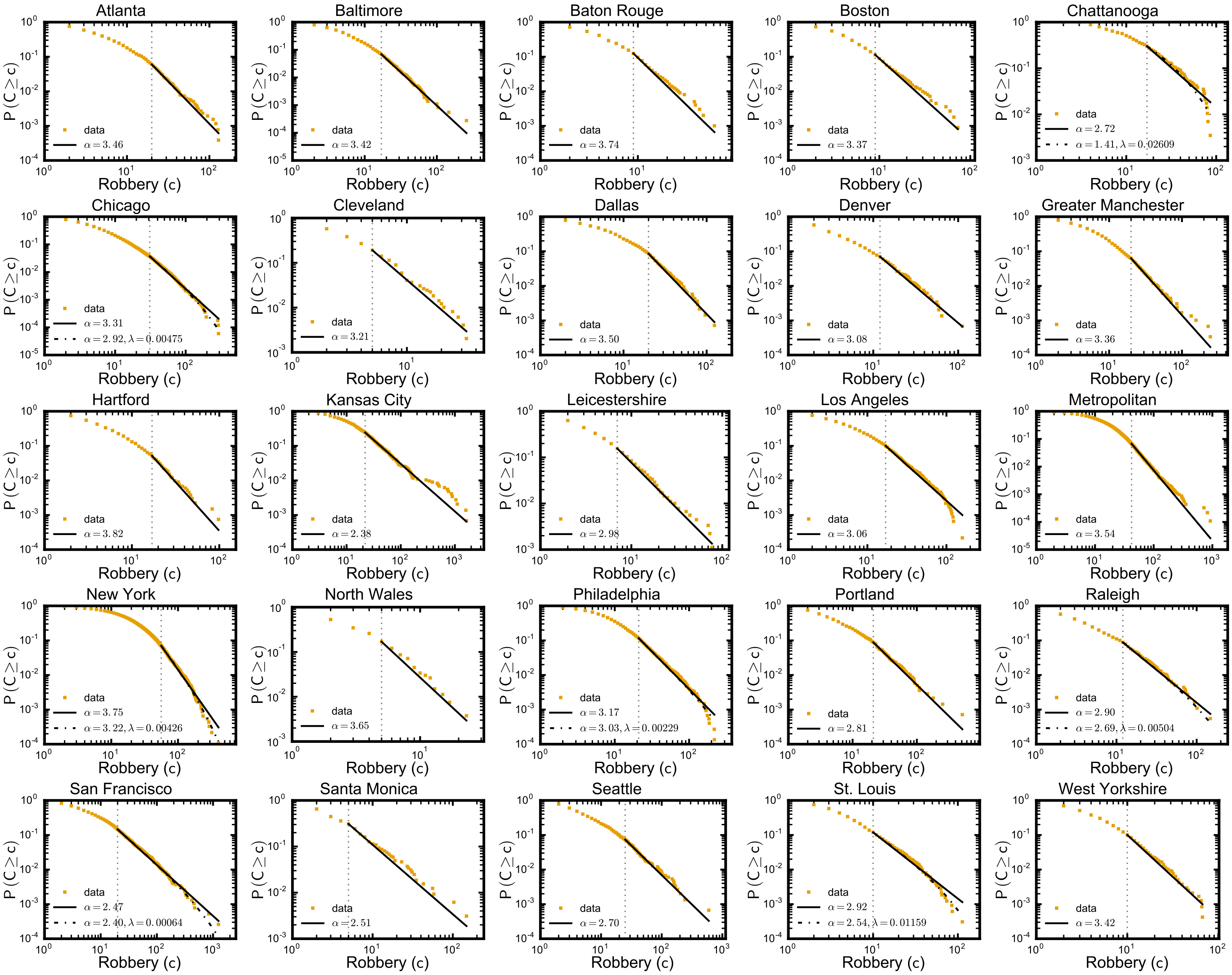

Figure 3: The best fits for the concentration of robbery in all considered cities.

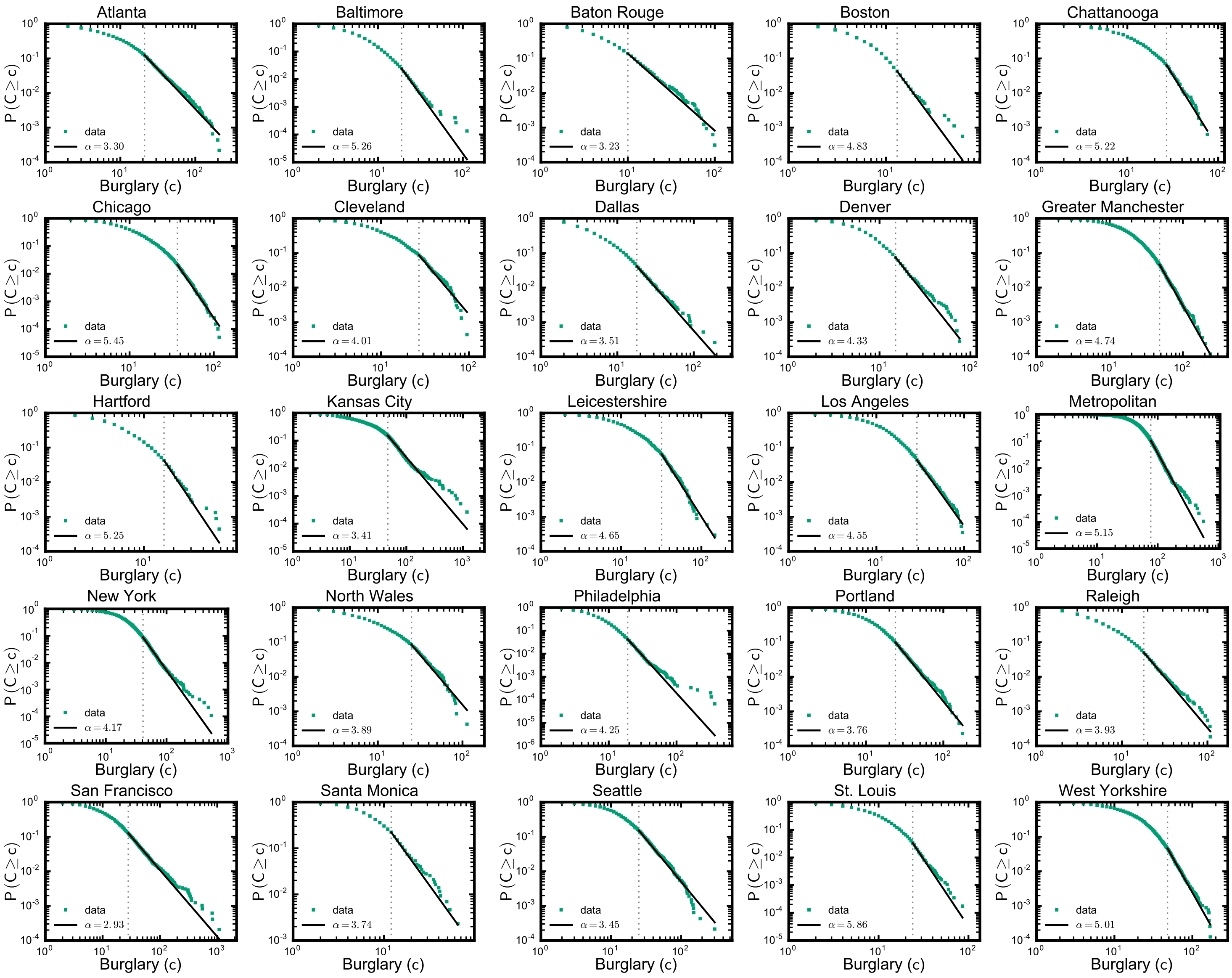

Figure 4: The best fits for the concentration of burglary in all considered cities.

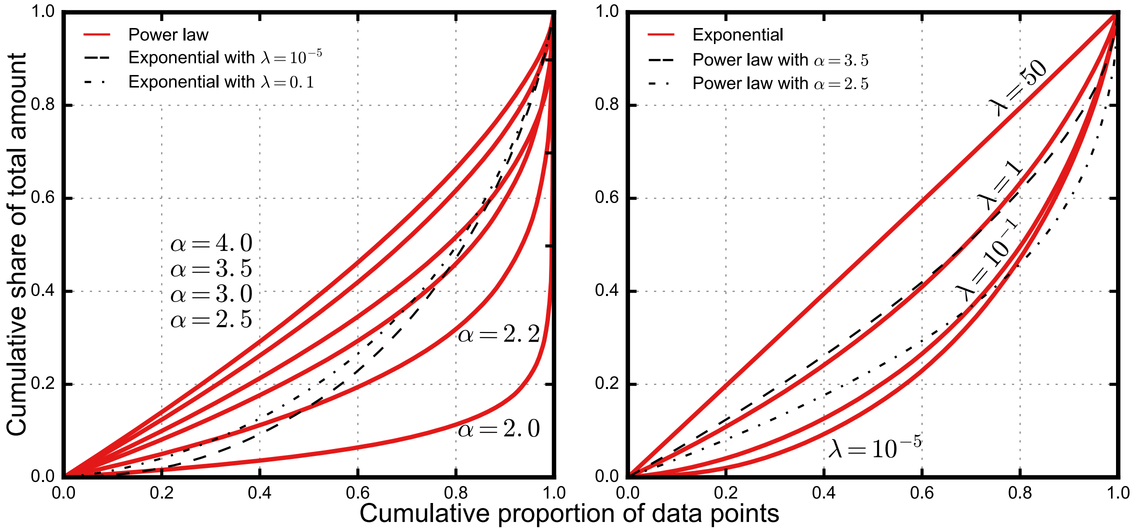

Figure 5: The distribution of a certain quantity following a power-law distribution with high α exponent yields concentrations similar to exponential distributions.

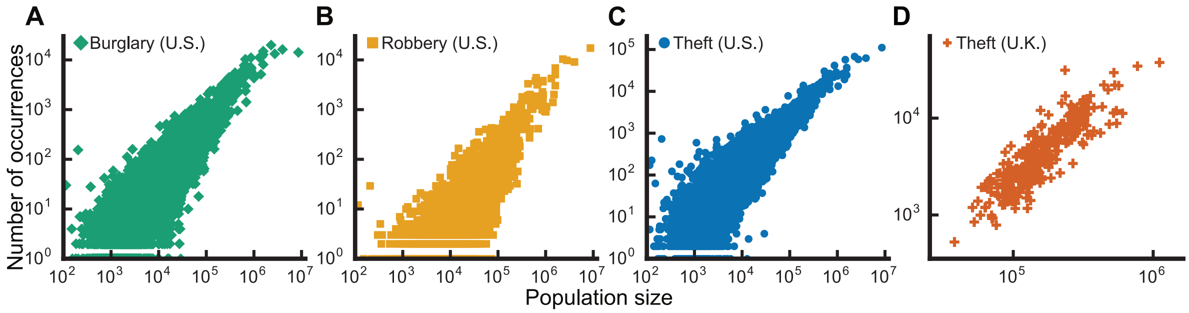

Figure 6: The way crime increases with the growth of the city relates to the type of crime. The amount of violent crimes in cities has been shown to scale superlinearly ($\beta \approx 1.15$) with city size [9, 10]. Scaling laws in cities, however, depend on the definition of the city as well as on the model for the fluctuations around population size N, that is, the conditional $\Pr(Y|N)$ [11, 12]. Intriguingly, criminal allometries relate also to the type of crime. For instance, (A) burglaries scale linearly with population size regardless of fluctuation model, whereas (B) robberies exhibit sublinearity or superlinearity depending on the model used. In the case of thefts, (C–D) we found superlinear increase with population size, independent of fluctuation model (see Table 10).

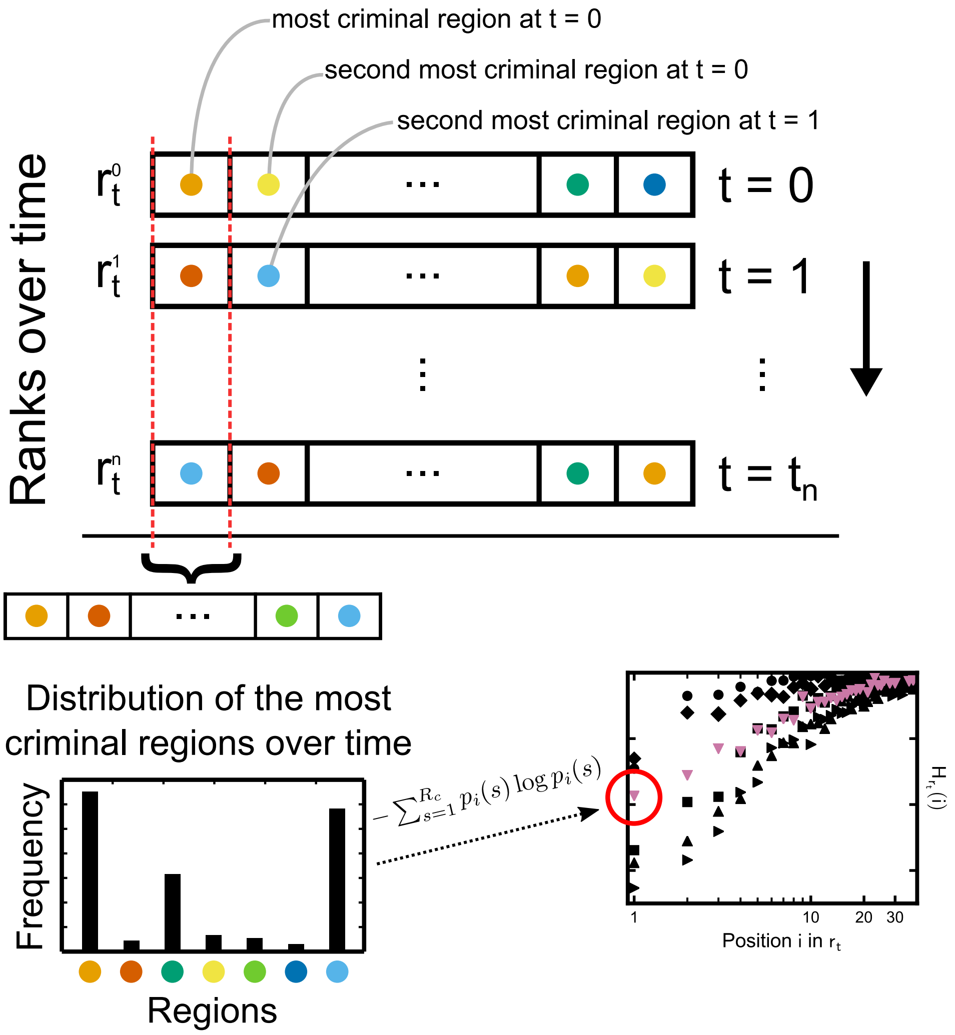

Figure 7: An example of measuring the entropy of a position in the rankings.

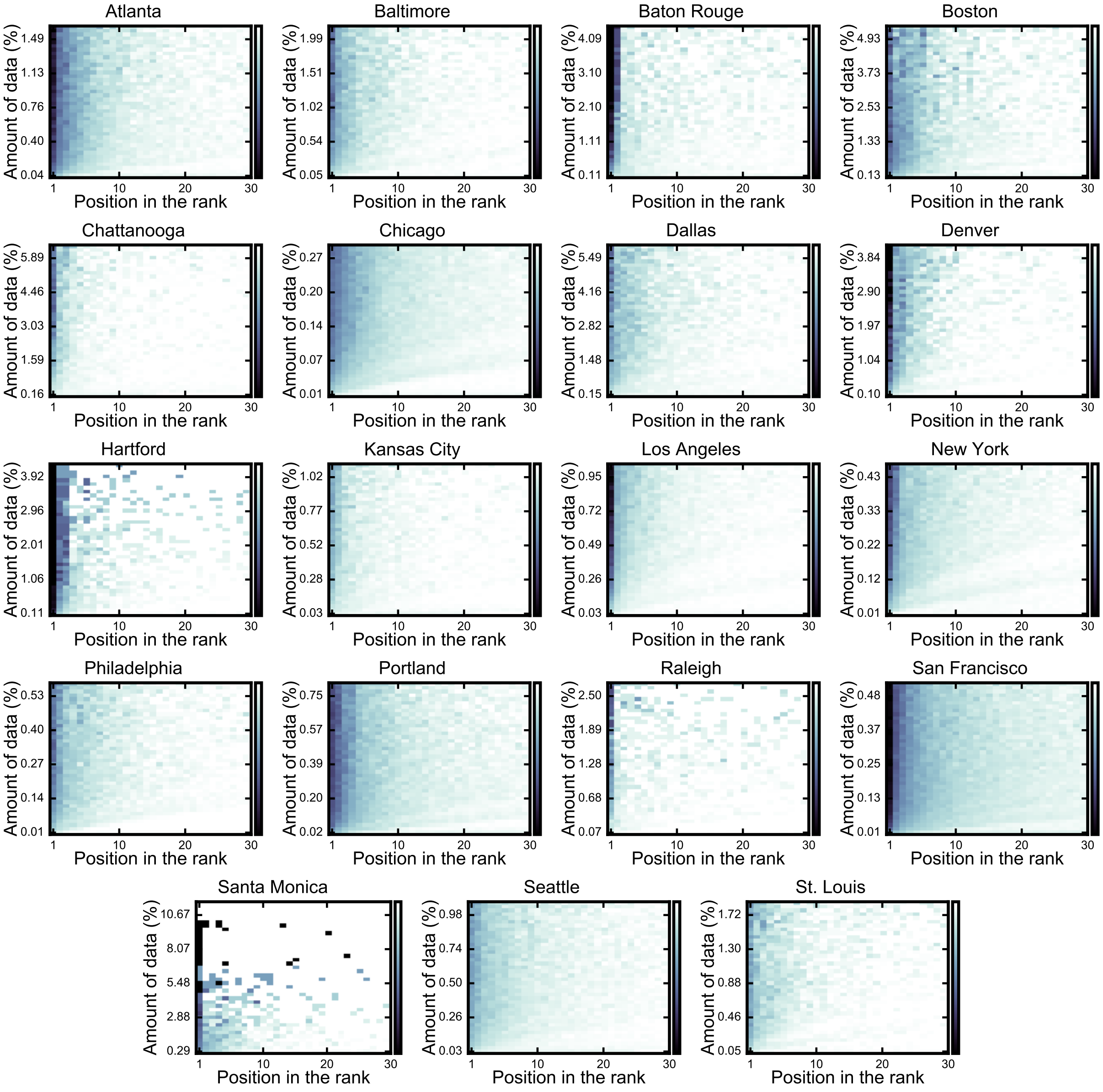

Figure 8: The influence of the amount of data a_w on the entropy of the positions in the rank of thefts H_a.
